# Early Adversity Imprints Multisystem Biological Aging: A Longitudinal Study of Adversity Dimensions and Aging Phenotypes Across Adolescence

**DOI:** 10.1016/j.bpsgos.2026.100782

**Published:** 2026-06-24

**Authors:** Chase Antonacci, Eugenia Giampetruzzi, Jessica L. Buthmann, Sarah Rocha, Xinyan Tao, Ian H. Gotlib

**Affiliations:** a Department of Psychology, Stanford University, Stanford, California; b Neurosciences Interdepartmental Program, Stanford University, Stanford, California

**Keywords:** Adolescence, Biological aging, Developmental imprinting, Developmental timing, Early-life adversity, Pubertal development

## Abstract

**Background:**

Early-life stress (ELS) is associated with accelerated biological aging; however, it is unclear whether dimensions of adversity such as threat and deprivation produce distinct multisystem aging phenotypes. Here we characterize, for the first time, the multivariate copatterning of stress dimensions and aging biomarkers and examine whether such phenotypes predict psychopathology 6 years later.

**Methods:**

In a longitudinal sample (*N* = 225; ages 9–13; 58.7% female) studied across 4 waves (approximately 6 years), 16 measures of stress exposure were reduced via principal component analysis to 3 orthogonal dimensions: cumulative stress & trauma, low parental support, and neighborhood disadvantage. Canonical correlation analysis (CCA) related these dimensions to baseline levels and developmental trajectories of 6 aging biomarkers (mitochondrial DNA [mtDNA], telomere length, cortisol, body mass index [BMI], pubertal stage, and brain age gap). Canonical variate (CV) scores predicted internalizing, externalizing, and total problems in late adolescence (*n* = 142).

**Results:**

CCA identified 2 significant CVs at baseline. CV1 linked deprivation-related stress (low parental support/neighborhood disadvantage) to a cellular-metabolic aging profile (higher BMI, older brain age, and lower mtDNA) (*r* = 0.39, *p* < .001). CV2 linked cumulative stress and trauma to a threat-responsive neuroendocrine maturation profile (earlier pubertal stage, higher cortisol, higher BMI, and higher mtDNA) (*r* = 0.24, *p* = .003). ELS was not associated with biomarker trajectories (*p*s > .20), consistent with early embedding. Only the threat phenotype (CV2) predicted psychopathology (β = 6.98, *p* = .004, *R*^2^ = 0.12).

**Conclusions:**

ELS is biologically embedded along adversity type-specific pathways detectable by late childhood, supporting a developmental imprinting model. A threat-related multisystem aging phenotype conferred transdiagnostic psychiatric risk in late adolescence, pointing to pre-adolescent development as an important window for prevention.

## Plain language summary

Children who experience early adversity face higher risk for accelerated biological aging and mental health problems, but whether distinct types of adversity produce different multisystem biological signatures is unknown. In a 6-year study of 225 children, we measured biological markers across cellular, metabolic, neuroendocrine, and brain systems alongside detailed stress assessments. Deprivation (e.g., low parental support) and threat (e.g., trauma exposure) each produced a distinct biological aging profile by early adolescence; only the threat-related profile predicted mental health problems 6 years later. These findings indicate that adversity type shapes biological development differently, with implications for targeted early intervention.

Early-life stress (ELS) is among the most potent and enduring environmental risk factors for psychopathology across the life span. Children who experience early stress have elevated rates of depression, anxiety, conduct problems, and substance use that persist well into adulthood (1,2). Furthermore, early adversity accounts for a substantial proportion of population-attributable risk for psychiatric disorders (3,4). Despite the strength and consistency of these associations, it remains unclear how distinct dimensions of stress exposure differentially shape coordinated patterns of functioning across biological systems, and how these multisystem configurations translate into lasting psychiatric risk (5). Identifying these mechanisms is essential not only for understanding how early experience shapes long-term development but also for pinpointing the biological targets and developmental windows that are most amenable to intervention.

Accelerated biological aging has emerged as a compelling mechanistic pathway linking early adversity to lasting health risk. Across multiple physiological systems, ELS has been associated with hallmarks of accelerated aging, including shorter telomeres (6), altered mitochondrial DNA (mtDNA) copy number (7), advanced brain age estimated from structural neuroimaging (8,9), earlier pubertal maturation (10), elevated body mass index (BMI) (11), and dysregulation of the hypothalamic-pituitary-adrenal (HPA) axis, reflected in altered patterns of cortisol secretion consistent with neuroendocrine programming and cumulative physiological wear (12,13). A meta-analysis synthesizing 54 studies (*N* = 116,010) found that ELS is associated with accelerated pubertal development (*d* = −0.26) and cellular aging (*d* = −0.43) in childhood and adolescence (14), with longitudinal work further demonstrating that stressful life events prospectively predict accelerated epigenetic aging and pubertal progression in youth over time (15). Critically, these markers of biological aging are not merely correlates of adversity; accelerated biological aging itself has been linked prospectively to risk for depression and anxiety, with a large-scale study of over 400,000 adults demonstrating that faster biological aging predicts incident internalizing disorders across nearly a decade-long follow-up (16). Together, these findings raise the possibility that biological aging represents a pathway through which ELS becomes translated into lasting psychiatric risk. However, to date, researchers have largely treated stress as a cumulative, global construct and have examined aging biomarkers individually, leaving unaddressed the question of whether distinct domains of adversity are linked to distinct, multisystem configurations of biological aging.

Theoretical frameworks in developmental psychopathology suggest that stress exposure is not monolithic in its biological effects. The Dimensional Model of Adversity and Psychopathology (17,18) draws a fundamental distinction between threat-based adversity (experiences involving direct harm or threat of harm, including abuse, violence, and trauma) and deprivation-based adversity (absence of expected environmental inputs, including neglect, poverty, and low parental support). These adversity types have been shown to differentially shape distinct neural circuits, with threat driving fear-learning and threat-detection systems and deprivation influencing circuits that support cognitive development and executive functioning (17,19). Importantly, this stress-type specificity appears to extend to biological aging. The meta-analytic findings of accelerated pubertal and cellular aging described above were specific to threat-based adversity and were not obtained for deprivation or low socioeconomic status (14). Furthermore, ELS has been shown to predict an accelerated cellular aging phenotype partly through earlier pubertal timing as a mediating pathway (20). Preliminary evidence in adults with major depression also suggests that threat and deprivation dimensions differentially predict specific biological aging markers (21). This prior work, however, examined adversity dimensions in relation to individual biomarkers rather than coordinated multisystem phenotypes, has not yet been extended to community samples of youth in prospective longitudinal designs, and has not tested whether biological aging configurations that are specific to different types of stress carry risk for the subsequent development of psychopathology.

The present study addresses these gaps using data from a longitudinal community sample of 225 youth followed across 4 assessment waves spanning approximately 6 years from late childhood through late adolescence. Stress exposure across 16 domains was decomposed via principal component analysis (PCA) into 3 orthogonal dimensions: cumulative stress and trauma, low parental support, and neighborhood disadvantage. Using canonical correlation analysis (CCA), we characterized the multivariate copatterning of these stress dimensions with 6 biological aging markers spanning cellular (i.e., mtDNA copy number and telomere length), metabolic (i.e., BMI), neuroendocrine (i.e., salivary cortisol area under the curve [AUC] and pubertal stage), and neurologic (i.e., brain age gap) systems, both at baseline and across developmental trajectories. Canonical variate (CV) scores were then used to predict levels of emotional and behavioral problems assessed 6 years later. We hypothesized that 1) distinct stress dimensions will be associated with distinct, coherent multisystem biological aging phenotypes that are consistent with the threat/deprivation distinction; 2) stress-biology associations will be stronger at baseline than in developmental trajectories, consistent with developmental imprinting rather than ongoing accumulation across adolescence; and 3) the biological aging phenotype associated with threat-related stress will confer prospective transdiagnostic risk for psychopathology in late adolescence.

## Methods and Materials

### Participants and Study Design

**Figure 1:**
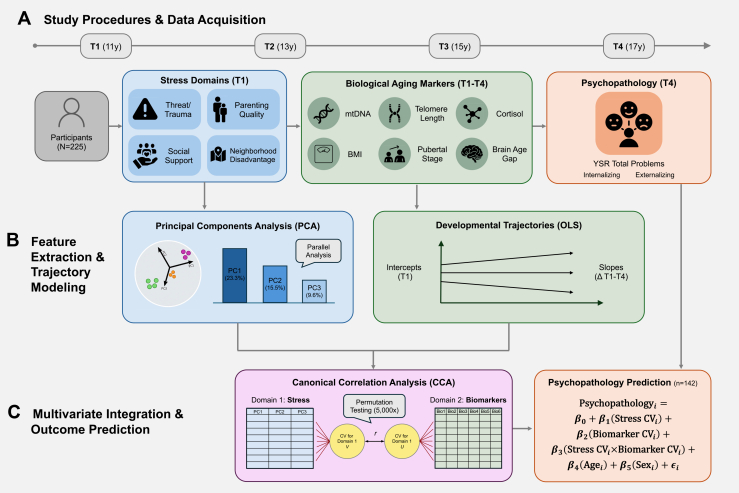
Study design and analytic overview. **(A)** Study procedures and data acquisition. Participants (*N* = 225) were assessed across 4 waves spanning early-to-late adolescence (T1: age approximately 11 years, T2: age approximately 13 years, T3: age approximately 15 years, and T4: age approximately 17 years). Early-life stress was characterized at T1 across 4 domains: cumulative stress & threat/trauma exposure, parenting quality, social support, and neighborhood disadvantage. Biological aging markers—including mitochondrial DNA (mtDNA) copy number, telomere length, salivary cortisol (diurnal area under the curve with respect to ground), body mass index (BMI), pubertal stage (Tanner), and brain age gap—were assessed longitudinally from T1 through T4. Psychopathology outcomes (Youth Self-Report [YSR] internalizing, externalizing, and total problems) were assessed at T4. **(B)** Feature extraction and trajectory modeling. Stress measures were reduced via PCA with parallel analysis retention; 3 components (PC1–PC3) were retained explaining 23.3%, 15.5%, and 9.6% of variance, respectively. Person-specific intercepts (T1 baseline level) and slopes (rate of change from T1–T4) were estimated for each biological aging marker via ordinary least squares regression, yielding 12 biomarker parameters as inputs to subsequent analyses. **(C)** Multivariate integration and outcome prediction. Canonical correlation analysis (CCA) simultaneously decomposed the stress PC matrix and aging biomarker matrices to identify latent dimensions of shared variance (canonical variates [CVs]); significance was evaluated via permutation testing (5000 permutations). CV scores were then used to prospectively predict psychopathology outcomes at T4 in covariate-adjusted linear regression models. OLS, ordinary least squares.

Participants were 225 youth (mean age = 11.43 years, SD = 1.06; 58.7% female) (Table 1) recruited from the San Francisco Bay area between 9 and 13 years of age for a longitudinal study examining the effects of ELS on psychobiological development. The study was approved by the Stanford University Institutional Review Board and conducted in compliance with all relevant laws and guidelines. Participants provided written informed assent/consent and were compensated for all study activities. Exclusion criteria included magnetic resonance imaging (MRI) contraindications, history of major mental or medical illness (e.g., major depressive disorder, epilepsy, or cardiovascular conditions), severe learning disabilities (e.g., attention, reading, or auditory processing problems), and non-fluency in English. At the baseline assessment (T1), participants completed 16 measures assessing lifetime exposure to stress and trauma across multiple domains. Beginning at T1 and continuing across 3 follow-up assessments (T2–T4) (Figure 1) conducted approximately 2 years apart, participants provided data on 6 biological aging markers: BMI, self-reported pubertal stage, mtDNA copy number, brain age gap (estimated via structural MRI), salivary cortisol AUC, and telomere length. At T4, a subset of participants (*n* = 142, 60.6% female; mean age = 17.69 years, SD = 1.38) completed the Youth Self-Report (YSR) (22), a widely used measure of adolescent psychopathology yielding scores for internalizing, externalizing, and total problems.

**Table 1:** Sample Characteristics (*N* = 225)

	Values			
Demographics				
Sex, Female/Male	132/93			
Age, Years				
Baseline, T1	11.43 (1.06) [9–13]			
2-year follow-up, T2	13.33 (1.12) [11–15]			
4-year follow-up, T3	15.60 (1.12) [13–18]			
6-year follow-up, T4 (*n* = 142)	17.69 (1.38) [14–21]			
Household Income-to-Needs Ratio	1.28 (0.56)			
Parent Education	4.88 (1.30)			
Race				
African American	19 (8.44%)			
Asian	24 (10.67%)			
Hispanic/Latino	20 (8.89%)			
Multiple races	46 (20.44%)			
Other	16 (7.11%)			
White	100 (44.44%)			
Psychopathology in Late Adolescence (T4)				
YSR total problems	44.79 (26.98)			
YSR internalizing problems	14.37 (10.16)			
YSR externalizing problems	11.24 (7.79)			

Stress PCs	Variance Explained			

PC1, Cumulative Stress & Trauma	22.6%			
PC2, Low Parental Support	14.9%			
PC3, Neighborhood Disadvantage	9.1%			

Aging Biomarkers	T1	T2	T3	T4

Body Mass Index	18.65 (3.67)	20.36 (4.03)	22.08 (4.60)	23.17 (4.74)
Pubertal Stage	2.02 (0.74)	3.47 (0.84)	4.32 (0.66)	4.71 (0.36)
Mitochondrial DNA	509.34 (165.97)	608.08 (203.71)	634.66 (222.26)	888.26 (925.36)
Brain Age Gap	0.36 (1.26)	−1.03 (1.07)	−2.24 (1.09)	−3.41 (1.31)
Cortisol AUC	1.29 (0.69)	2.12 (1.03)	3.29 (2.28)	2.85 (1.56)
Telomere Length	1.5 (0.25)	1.37 (0.22)	1.2 (0.18)	1.16 (0.16)

Values are presented as *n*, *n* (%), mean (SD) [range], or mean (SD). Household income-to-needs ratio and parent education coding details are available in the Supplement.

AUC, area under the curve; PC, principal component; T, time; YSR, Youth Self-Report.

### Stress Measures

ELS was assessed via 16 measures spanning 4 domains: threat/trauma [Traumatic Events Screening Inventory for Children (23) and the Early Life Stress Questionnaire (24)]; parenting quality [neglect (25), authority (26), criticism (27), monitoring (28), psychological control (29,30), warmth (31), and parent psychopathology (32)]; perceived social support [Multidimensional Scale of Perceived Social Support (33)]; and neighborhood disadvantage [pollution burden and population characteristics subscales from the CalEnviroScreen version 4.0 (34) and the Area Deprivation Index (35,36)]. All measures were scored such that higher values reflected greater adversity. Full details on each measure, psychometric properties, and administration procedures are provided in the Supplement.

### Biomarkers of Aging

Six markers spanning cellular, metabolic, neuroendocrine, and neurologic systems were assessed longitudinally. Cellular aging was indexed by mtDNA copy number and telomere length (T/S ratio), both from salivary DNA. Neuroendocrine activity was indexed by salivary cortisol assessed via diurnal sampling (after waking, 30 minutes post-waking, in the mid-afternoon, and in the evening) and summarized as AUC with respect to ground. Somatic development was captured by BMI and self-reported pubertal stage (Tanner staging). Finally, neurobiological aging was indexed via brain age gap, the difference between a machine learning-predicted brain age derived from structural MRI and chronological age, residualized for age-related bias. Full details on assay procedures, image acquisition, and brain age estimation are provided in the Supplement.

### Outcome Measures

Psychopathology was assessed at T4 (approximately 6 years post-baseline) using the YSR, a 112-item self-report measure of emotional and behavioral functioning (22). YSR items were aggregated into internalizing, externalizing, and total problems broadband scales, which were treated as continuous outcomes. The YSR demonstrates good internal consistency (α = 0.71–0.91) and test–retest reliability (*r* = 0.78–0.89) (22, 37).

### Statistical Analyses

All analyses were implemented in Python version 3.10.12, and permutation tests used a fixed seed (seed = 42) for reproducibility.

Biomarker missingness was low at baseline (10%) and increased across later waves because of participant attrition and assay availability, averaging 36% across all variables and time points. Stress dimensions did not significantly predict missingness for any biomarker (all *p*s > .16), supporting a missing at random assumption. Missing values were handled via multiple imputations by chained equations (m = 10 imputed datasets, 10 iterations) (38) using predictive mean matching. Pooled estimates were obtained by averaging across imputed datasets (39,40). All primary analyses used the pooled sample (*N* = 225).

Stress exposure variables were submitted to PCA via singular value decomposition to reduce collinearity before CCA. Because individual stress exposures tend to be moderately intercorrelated (41,42), entering all 16 variables directly into the CCA would introduce collinearity, reducing solution stability and inflating the risk of overfitting given the sample size. Therefore, we used PCA to first reduce the predictor set to a smaller number of orthogonal dimensions capturing the major axes of stress covariance. Parallel analysis (1000 permutations; 95th percentile) supported retaining 3 components in unrotated form: PC1 (cumulative stress and trauma, 23.3% of variance), PC2 (low parental support, 15.5%), and PC3 (neighborhood disadvantage, 9.6%). See the Supplement for PC loadings.

For each participant, baseline biomarker values (intercepts) were taken as observed T1 scores. Individual developmental trajectories (slopes) were estimated via ordinary least squares (OLS) regression of each biomarker on chronological age across T1 through T4. Person-specific OLS slopes were preferred over random-effects estimates to preserve individual variability in trajectories for use in subsequent multivariate analyses because shrinkage toward the group mean would limit trajectory estimation for biomarkers that follow relatively stereotyped developmental progressions (e.g., brain age), where even small individual deviations from normative trajectories are theoretically meaningful (43).

To characterize the multivariate structure of associations between stress dimensions and biological aging, we used CCA, which identifies linear combinations of stress dimensions and biological aging markers that maximally covary, thereby revealing emergent multisystem biological phenotypes that would be difficult to detect using univariate approaches. We conducted CCA using singular value decomposition of the cross-covariance matrix between standardized stress PC scores (X; 3 variables) and standardized biomarker values (Y; 6 variables), separately for biomarker intercepts and slopes. Statistical significance of each CV was assessed via permutation testing (5000 permutations). For each permutation, rows of the biomarker matrix Y were randomly shuffled while holding the stress-PC matrix X fixed, preserving the within-matrix covariance structures while breaking any true X-Y association. Each variate was tested independently by comparing the observed canonical correlation against the distribution of null values from the same ordinal position, with *p* equal to the proportion of null values meeting or exceeding the observed value (44) (minimum *p* = .0002). Null distributions and observed values for each variate are visualized in Figure S4.

To decompose canonical effects into biomarker-specific associations, each biomarker intercept (and slope in sensitivity analyses) was simultaneously regressed on PC1, PC2, and PC3 in separate OLS models. Regression coefficients were standardized by the biomarker SD to facilitate cross-biomarker comparison, and significance was assessed via permutation testing (2000 permutations of the outcome vector). These models did not include age or sex as covariates, consistent with the primary CCA, as they are intended as confirmatory decomposition of the canonical structure rather than independent hypothesis tests. Furthermore, to assess the developmental patterning of stress-biomarker associations, cross-sectional Pearson correlations between stress PC scores and biomarker values were computed at each wave (T1–T4), with attenuation quantified as percent change in the absolute correlation from T1 to T4.

Finally, CV scores from the intercepts model were examined as predictors of YSR outcomes at T4 via linear regression, with the stress and biomarker CV scores, their interaction, age, and sex as predictors. YSR total problems was specified a priori as the primary outcome. Given a significant effect on total problems, post hoc models examined YSR internalizing and externalizing problems as secondary outcomes. Bivariate associations between CV scores and outcomes are reported as descriptive context. These analyses were conducted in the subset of participants with T4 YSR data available (*n* = 142).

## Results

### Participant Characteristics

The analytic sample comprised 225 participants (mean age = 11.43 years, SD = 1.06; 58.7% female) (Table 1) with usable stress and biomarker data. The subset of participants with T4 YSR data available for outcome modeling (*n* = 142, 60.6% female; mean age at T4 = 17.69 years, SD = 1.38) reported mild to moderate levels of internalizing (mean = 14.37, SD = 10.16), externalizing (mean = 11.24, SD = 7.79), and total (mean = 44.79, SD = 26.98) problems in late adolescence. Participants with T4 outcome data (*n* = 142) did not differ significantly from those without T4 data (*n* = 83) on any dimension of stress exposure (all *p*s > .35).

### CCA: Stress Dimensions and Biological Aging

**Figure 2:**
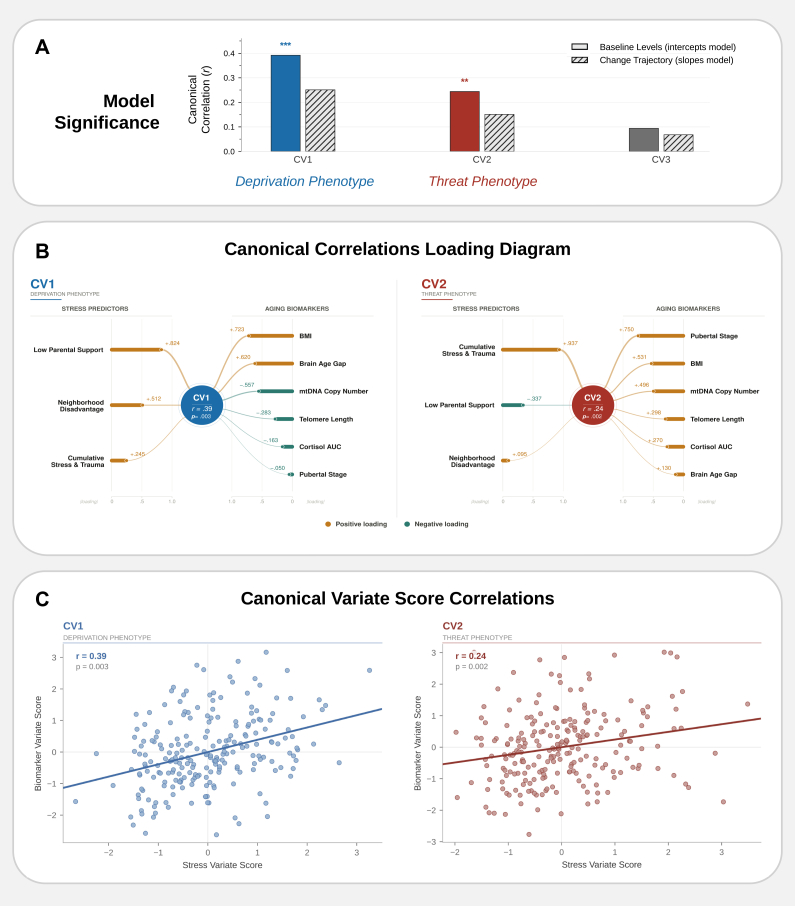
Canonical correlation analysis of early-life adversity and biological aging markers. Two adversity-type-specific canonical variates (CVs) emerged from baseline biomarker levels but not from developmental trajectories, indicating that early-life stress imprints stable biological aging profiles that are already detectable by early adolescence rather than driving ongoing dysregulation across development. **(A)** Canonical correlations for the intercepts model (baseline biomarker levels; solid bars) and slopes model (developmental trajectories; hatched bars) for each canonical variate. CV1 and CV2 were significant in the intercepts model (permutation testing, 5000 permutations); no variate reached significance in the slopes model. **(B)** Variable loading diagrams for CV1 (deprivation phenotype, blue) (*r* = 0.39, *p* = .003) and CV2 (threat phenotype, red; *r* = 0.24, *p* = .002) from the intercepts model. CV1 is characterized by deprivation-related stressors (low parental support, neighborhood disadvantage) predicting a cellular-metabolic aging profile (elevated body mass index [BMI], older brain age gap, and reduced mitochondrial DNA [mtDNA] copy number). CV2 is characterized by cumulative stress & trauma predicting a pubertal-neuroendocrine profile (earlier pubertal timing, elevated BMI, and greater mtDNA copy number). **(C)** Canonical variate score correlations for CV1 (left) and CV2 (right). Each point represents one participant (*N* = 225); line indicates ordinary least squares fit. AUC, area under the curve.

CCA of baseline biomarkers identified 2 significant CVs (Figure 2A). CV1 (*r* = 0.392, *p* < .001) (Figure 2C) was characterized by strong positive loadings for low parental support (+0.82) and neighborhood disadvantage (+0.51), and on the biomarker side by higher BMI (+0.72), older brain age gap (+0.62), and lower mtDNA copy number (−0.56) (Figure 2B). Together, these loadings reflect a cellular-metabolic aging phenotype consistent with cumulative physiological attrition under chronic resource scarcity. In contrast, CV2 (*r* = 0.244, *p* = .003) (Figure 2C) was dominated by cumulative stress and trauma (+0.94). It indexed a coordinated pattern of somatic maturation and neuroendocrine upregulation: earlier pubertal stage (+0.75), higher BMI (+0.53), elevated mtDNA copy number (+0.50), longer telomere length (+0.30), and higher cortisol secretion (+0.27) (Figure 2B). This pattern reflects threat-responsive neuroendocrine mobilization, consistent with a physiological recalibration adaptive to persistent threat within life-history theory frameworks. Notably, mtDNA was reduced in CV1 but elevated in CV2, consistent with mitochondrial biogenesis under heightened neuroendocrine demand rather than the cellular attrition characteristic of deprivation.

The third variate was not statistically significant (*r* = 0.094, *p* = .310) (Figure 2A), and the parallel slopes model CCA did not yield any significant variates (all *p*s > .28) (Figure 2A). This dissociation is consistent with a developmental imprinting model, in which adversity shapes biological aging setpoints detectable at study entry rather than driving continued dysregulation across adolescence.

### Simultaneous Multiple Regression: Biomarker-Specific Associations

**Figure 3:**
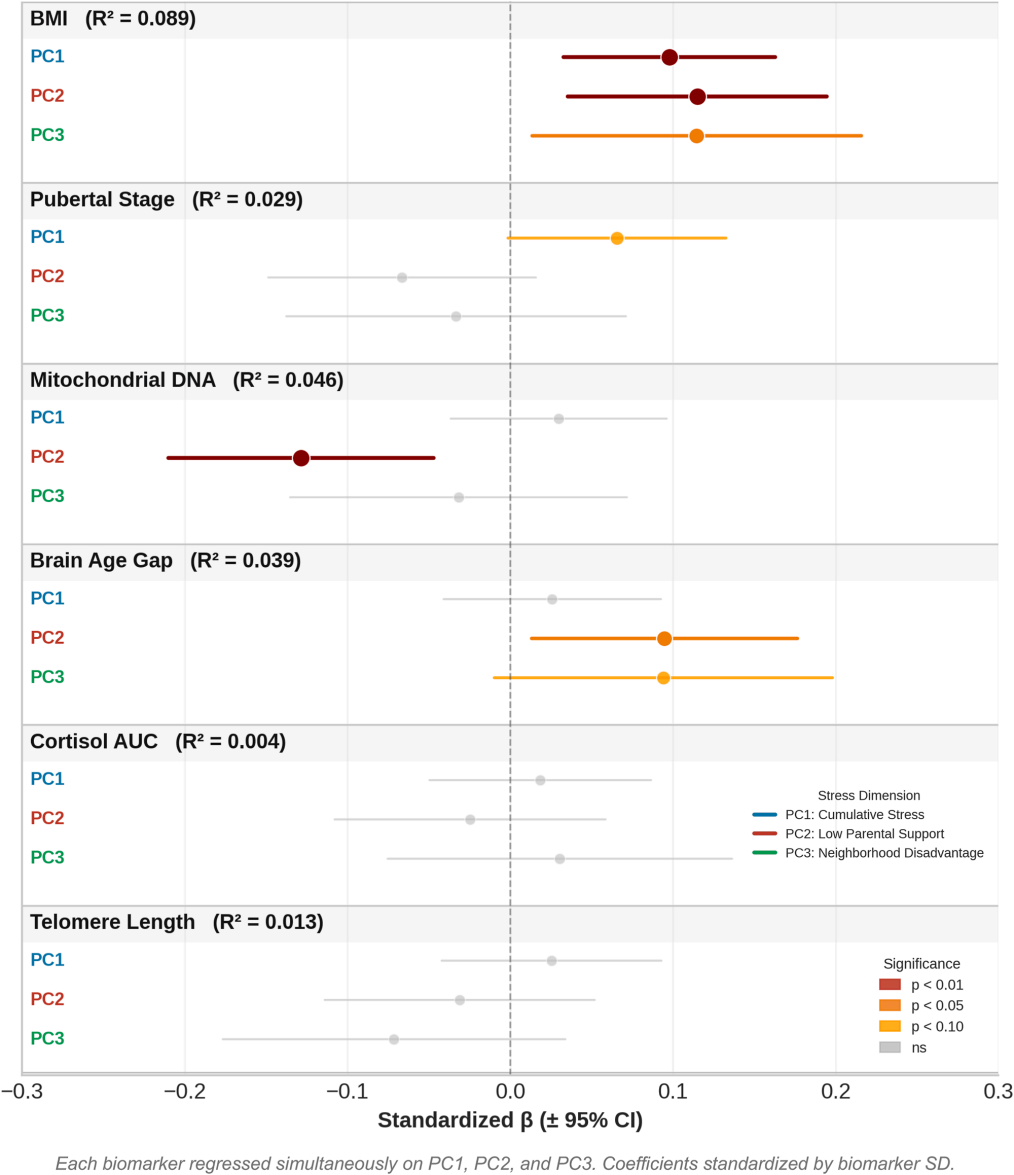
Stress-type-specific associations between early-life adversity dimensions and biological aging markers at baseline. Forest plot of simultaneous multiple regression results predicting each baseline biological aging marker from all 3 stress dimensions jointly (*N* = 225). Deprivation-related stress (PC2, PC3) is the primary driver of cellular aging dysregulation—specifically reduced mitochondrial DNA copy number and elevated brain age gap—whereas all 3 stress dimensions independently predict higher body mass index (BMI). Points represent standardized regression coefficients (β); error bars represent 95% CIs. Point color indicates significance (permutation *p* values). *R*^2^ for each biomarker model is shown in the header row. AUC, area under the curve; PC, principal component.

**Figure 4:**
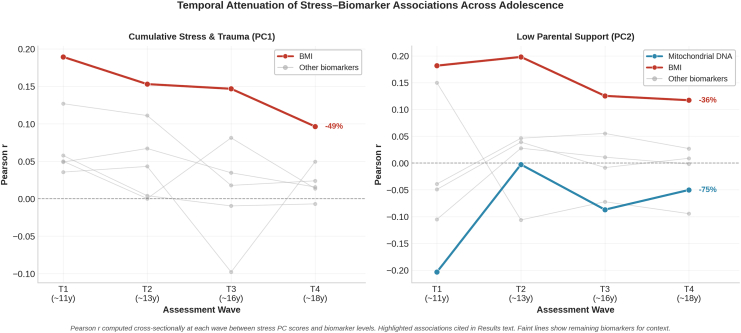
Attenuation of stress-biomarker associations across development. Cross-sectional Pearson correlations between stress principal component (PC) scores and biomarker levels computed separately at each assessment wave (T1–T4; *N* = 225). Stress-biomarker associations present in early adolescence weaken substantially over 6 years, consistent with biological embedding at early developmental setpoints rather than ongoing stress-driven accumulation. Left: cumulative stress & trauma (PC1). Right: low parental support (PC2). Bold colored lines highlight associations discussed in the Results: PC1 × body mass index (BMI) (−49% from T1 to T4), PC2 × mitochondrial DNA (−75%), and PC2 × BMI (−36%). Faint gray lines show remaining biomarker associations for context. Annotated percentages reflect the proportional change in Pearson's *r* from T1 to T4.

To decompose canonical effects into biomarker-specific associations, each baseline biomarker was simultaneously regressed on all 3 stress PCs (Figure 3). BMI showed the strongest associations (*R*^2^ = 0.089), with all 3 stress dimensions independently predicting greater BMI (PC1: β = 0.098, *p* = .004; PC2: β = 0.115, *p* = .006; PC3: β = 0.115, *p* = .026). Low parental support (PC2) independently predicted lower mtDNA copy number (β = −0.129, *p* = .004, *R*^2^ = 0.046) and older brain age (β = 0.095, *p* = .022, *R*^2^ = 0.039). Marginal associations emerged for cumulative stress and trauma predicting earlier pubertal stage (PC1: β = 0.066, *p* = .061) and neighborhood disadvantage predicting older brain age (PC3: β = 0.094, *p* = .076). Although cortisol and telomere length showed no independent associations with any stress dimension (*p*s > .19), this does not preclude their contribution to the canonical solution. Variables may load meaningfully on CVs through shared covariance with other biomarkers rather than through direct univariate effects. Parallel slopes models were nonsignificant (*p*s > .05), consistent with the null slopes CCA. Temporal attenuation analyses confirmed that stress-biomarker associations were strongest at baseline and attenuated across waves: low parental support-mtDNA declined from *r* = −0.203 (T1) to *r* = −0.050 (T4) (−75.4%), and low parental support-BMI from *r* = 0.182 (T1) to *r* = 0.117 (T4) (−35.7%) (Figure 4).

### Deprivation and Threat Phenotype Scores and Adolescent Psychopathology

**Figure 5:**
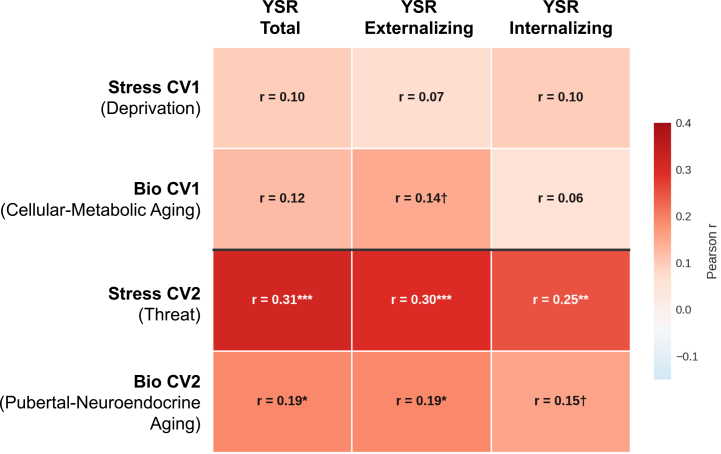
Bivariate associations between canonical variate (CV) scores and psychopathology in late adolescence. Pearson correlations between CV scores derived from the baseline intercepts model and Youth Self-Report (YSR) internalizing, externalizing, and total problems subscales assessed at T4 (*n* = 142). The threat phenotype (CV2), but not the deprivation phenotype (CV1), prospectively predicted psychopathology across all 3 symptom dimensions 6 years later, demonstrating adversity-type specificity in psychiatric risk. The bold horizontal line separates the CV1 (deprivation) and CV2 (threat) canonical pairs. CV2 stress scores (threat) were significantly associated with all 3 outcome indices; CV2 biomarker scores (pubertal-neuroendocrine aging) showed significant or trend-level associations with total problems and externalizing. Neither CV1 (deprivation) score was significantly associated with any psychopathology outcome. †*p* < .10, ∗*p* < .05, ∗∗*p* < .01, and ∗∗∗*p* < .001.

Next, we examined CV scores as prospective predictors of psychopathology 6 years later. Critically, the 2 CVs dissociated in their prospective associations. The deprivation phenotype (CV1) scores were not related to any T4 outcome (all | *r*s |< 0.16, *p*s > .08) (Figure 5). In contrast, the threat phenotype (CV2) stress scores were significantly associated with all 3 outcomes: total (*r* = 0.315), externalizing (*r* = 0.295), and internalizing (*r* = 0.250) problems (all *p*s < .003). Threat phenotype biomarker scores also showed significant bivariate associations with total problems (*r* = 0.191, *p* = .023) and externalizing problems (*r* = 0.190, *p* = .021), and a marginal association with internalizing problems (*r* = 0.154, *p* = .067), indicating that both components of the threat variate—the pattern of stress exposure and the biological phenotype it coorganizes—independently track risk for adolescent psychopathology.

In covariate-adjusted regression models (age and sex), CV pairs were jointly entered with their interaction, with YSR total problems specified a priori as the primary outcome. The deprivation phenotype (CV1) model was nonsignificant overall (*R*^2^ = 0.037, all *p*s > 0.15). The threat phenotype (CV2) model accounted for 12.3% of variance in total problems (*R*^2^ = 0.123); the stress component was the only significant predictor (β = 6.978, standard error [SE] = 2.361, 95% CI [2.350–11.605], *p* = .004). The biomarker component (*p* = .113) and stress × biomarker interaction (*p* = .894) were nonsignificant, suggesting parallel rather than synergistic pathways of risk. This attenuation is expected given CCAs covariance-maximizing construction (canonical *r* = 0.244). In joint models, the stress CV absorbs shared variance, leaving the biomarker CV with only its residual unique contribution. The bivariate associations may therefore provide a more direct characterization of each component’s independent relation to psychopathology.

Finally, post hoc models examined specificity across symptom dimensions. Threat phenotype stress scores independently predicted both externalizing (β = 2.197, SE = 0.688, 95% CI [0.848–3.545], *p* = .0005) and internalizing (β = 1.870, SE = 0.891, 95% CI [0.124–3.615], *p* = .039) problems, with biomarker scores and stress × biomarker interactions nonsignificant in both models. These findings indicate that transdiagnostic psychopathology risk is transmitted specifically by the threat phenotype and not the deprivation phenotype.

## Discussion

The present study examined whether distinct dimensions of ELS are associated with distinct patterns of biological aging in adolescence, and whether these stress-biology configurations predict subsequent psychopathology. Using CCA, we identified 2 significant multivariate associations between dimensions of stress exposure and markers of biological aging in late childhood. The deprivation phenotype (CV1) linked low parental support and neighborhood disadvantage to elevated BMI, accelerated brain age, and reduced mtDNA copy number. The threat phenotype (CV2) linked cumulative stress and trauma to earlier pubertal timing, elevated cortisol, higher BMI, and elevated mtDNA copy number. Critically, stress exposure was not associated with developmental trajectories of biomarkers; instead, stress-biomarker associations were concentrated at baseline and attenuated substantially across subsequent waves. This temporal pattern supports a developmental imprinting model in which adversity shapes biological setpoints during early sensitive periods rather than driving continued deterioration across adolescence. Finally, only the threat-related phenotype prospectively predicted transdiagnostic psychopathology 6 years later.

These findings extend dimensional models of adversity to encompass coordinated multisystem biological aging. Importantly, both deprivation sub-components loaded exclusively onto CV1 and the cumulative trauma dimension onto CV2, confirming that qualitatively distinct forms of adversity are associated with fundamentally different multisystem biological aging profiles (a finding replicated using theory-driven threat and deprivation composites; see the Supplement). The deprivation phenotype maps onto theoretical accounts of allostatic load (45,46), in which sustained energetic insufficiency produces physiological attrition across metabolic, neurologic, and cellular systems. Consistent with this formulation, simultaneous regression revealed that all 3 stress dimensions independently predicted elevated BMI, identifying metabolic dysregulation as the most convergent biological signature of adversity exposure. However, it is important to note that this reflects a population-level statistical association. Elevated BMI in an individual child should not be taken as evidence of adversity exposure, nor should adversity be expected to lead to elevated BMI in any given child. We also note that at these ages, elevated BMI should not be interpreted as a direct metric of biological aging itself; rather, adversity-linked BMI elevations in this developmental window are best understood as early indicators of metabolic dysregulation that prospectively predict cardiovascular and metabolic aging risk in adulthood (47,48). In contrast, the threat phenotype reflects a distinct profile of coordinated somatic acceleration: earlier pubertal timing, elevated cortisol, higher BMI, and greater mtDNA copy number. Within life history theory frameworks (49,50), this profile is posited to reflect a system-wide recalibration of developmental tempo and threat sensitivity, an adaptive response to persistent environmental danger. The opposing directions of mtDNA copy number across phenotypes are particularly informative. Under threat, elevated mtDNA is thought to reflect mitochondrial biogenesis compensating for heightened energetic demands of sustained neuroendocrine activation (51,52), whereas under deprivation, reduced mtDNA may reflect mitochondrial attrition driven by chronic oxidative stress (52,53). This dissociation highlights the importance of characterizing biomarkers within their multivariate adversity context rather than as isolated indicators of aging.

The attenuation of stress-biomarker associations across adolescence, coupled with the absence of stress-trajectory associations, suggests biological stabilization following early embedding rather than continued stress-driven dysregulation (54, 55, 56). This pattern is consistent with findings from animal studies demonstrating that early adversity produces stable epigenetic and neuroendocrine alterations that persist independent of continued stress exposure (57). Notably, telomere length, a marker for which between-person variation is largely established early in life (58,59), showed modest baseline loadings and near-zero trajectory associations, consistent with this early-embedding interpretation. Human epidemiological studies similarly show that early socioeconomic disadvantage predicts adult biological aging markers independent of adult socioeconomic conditions (60), suggesting that biological embedding occurs early and is not fully reversed by later environmental conditions. An alternative (though not mutually exclusive) explanation is that normative adolescent development accounts for substantial variance in biomarker trajectories that obscures early stress-related effects, particularly given our modest sample size. Importantly, this interpretation does not minimize the clinical significance of the stress-linked alterations identified at baseline, but rather locates their origins in early development, with implications for identifying optimal windows for prevention and intervention.

The prospective association between the threat-related phenotype and psychopathology parallels neuroimaging findings in which threat, but not deprivation, preferentially shapes threat-reactive neural circuitry linked to heightened emotional reactivity and broad psychopathology risk (19,41). The transdiagnostic nature of this effect, spanning internalizing, externalizing, and total problems, suggests operation through broad neurobiological mechanisms such as heightened arousal or emotional reactivity rather than symptom-specific pathways (61,62). Multiple complementary biological pathways likely contribute; earlier pubertal timing is a robust prospective risk factor for adolescent psychopathology (63,64), and chronic HPA upregulation impairs negative affect regulation and behavioral control (12).

In the joint regression model, the biomarker component of the threat phenotype did not independently predict psychopathology after accounting for stress, despite the significant bivariate association. This attenuation likely reflects mathematical coupling inherent to CCA, which maximizes covariance by construction, rather than indicating a null biological effect. Whether this phenotype mediates the stress-psychopathology link or instead represents a correlated marker of a shared underlying process cannot be determined from this correlational design. Establishing causal pathways will require formal mediation testing with temporally separated assessments or, ideally, intervention studies targeting stress exposure or biological aging processes.

We should note 5 limitations of this study. First, although the analytic sample was adequate for detecting 2 primary CVs, the modest size of our sample recruited from a single site may have limited our power to detect weaker associations and constrained generalizability. These findings should be replicated in larger and more diverse cohorts incorporating additional biomarkers (e.g., epigenetic clocks and inflammatory cytokines). Second, biomarker trajectories were estimated from 4 time points using person-specific OLS regression. This approach assumes linearity and may also introduce noise, which may have contributed to the null slopes CCA finding. Third, stress dimensions were assessed at a single baseline time point via retrospective self-report, which does not capture cumulative adversity across development. Prospective or longitudinal stress assessments may reveal stronger associations with developmental changes in biomarkers. Fourth, stress and biomarker CV scores share variance by mathematical construction, which limits the interpretability of their independent effects in joint regression models. Studies using longitudinal mediation designs or stress-reduction interventions are necessary to establish the causal structure of these associations. Finally, the present analyses are limited in their ability to speak to specific mechanisms underlying the identified stress-biomarker associations because multiple overlapping pathways are plausible for each biomarker (e.g., the BMI-adversity association may reflect food insecurity, reduced physical activity, or neuroendocrine metabolic consequences; cellular aging effects may involve oxidative stress, glucocorticoid exposure, or epigenetic modification). Studies measuring these processes directly are needed to determine whether distinct adversity types shape biomarkers through shared or system-specific pathways.

Despite these limitations, this study demonstrates that ELS becomes biologically embedded along stress-type-specific pathways that are detectable across multiple physiological systems by late childhood or early adolescence. We found that deprivation and threat, 2 qualitatively distinct dimensions of adversity, recruited fundamentally distinct biological responses: cellular-metabolic attrition on one pathway and coordinated somatic acceleration with neuroendocrine mobilization on another. These alterations reflect stable changes in biological baselines that are consistent with developmental imprinting. Furthermore, the threat-related phenotype confers lasting transdiagnostic risk for psychopathology spanning both internalizing and externalizing symptoms. Taken together, these findings underscore the formulation that characterizing adversity by type, and not merely quantifying exposure, is essential to understanding how early stress becomes biologically embedded and why it carries lasting consequences for mental health.

## Supplementary Material

Supplement Methods, Results, Figures S1–S5, and Tables S1–S3

Key Resources Table

## References

[1] Wade M., Wright L., Finegold K.E. (2022). The effects of early life adversity on children’s mental health and cognitive functioning. Transl Psychiatry.

[2] Kessler R.C., McLaughlin K.A., Green J.G., Gruber M.J., Sampson N.A., Zaslavsky A.M. (2010). Childhood adversities and adult psychopathology in the WHO World Mental Health Surveys. Br J Psychiatry.

[3] Green J.G., McLaughlin K.A., Berglund P.A., Gruber M.J., Sampson N.A., Zaslavsky A.M., Kessler R.C. (2010). Childhood adversities and adult psychiatric disorders in the national comorbidity survey replication I: Associations with first onset of DSM-IV disorders. Arch Gen Psychiatry.

[4] McLaughlin K.A., Greif Green J., Gruber M.J., Sampson N.A., Zaslavsky A.M., Kessler R.C. (2012). Childhood adversities and first onset of psychiatric disorders in a national sample of US adolescents. Arch Gen Psychiatry.

[5] Berens A.E., Jensen S.K.G., Nelson C.A. (2017). Biological embedding of childhood adversity: From physiological mechanisms to clinical implications. BMC Med.

[6] Ridout K.K., Levandowski M., Ridout S.J., Gantz L., Goonan K., Palermo D. (2018). Early life adversity and telomere length: A meta-analysis. Mol Psychiatry.

[7] Tyrka A.R., Parade S.H., Price L.H., Kao H.T., Porton B., Philip N.S. (2016). Alterations of mitochondrial DNA copy number and telomere length with early adversity and psychopathology. Biol Psychiatry.

[8] Beck D., Whitmore L., MacSweeney N., Brieant A., Karl V., de Lange A.G. (2025). Dimensions of early-life adversity are differentially associated with patterns of delayed and accelerated brain maturation. Biol Psychiatry.

[9] Drobinin V., Van Gestel H., Helmick C.A., Schmidt M.H., Bowen C.V., Uher R. (2022). The developmental brain age is associated with adversity, depression, and functional outcomes among adolescents. Biol Psychiatry Cogn Neurosci Neuroimaging.

[10] Ho T.C., Buthmann J., Chahal R., Miller J.G., Gotlib I.H. (2024). Exploring sex differences in trajectories of pubertal development and mental health following early adversity. Psychoneuroendocrinology.

[11] Morris T.T., Northstone K., Howe L.D. (2016). Examining the association between early life social adversity and BMI changes in childhood: A life course trajectory analysis. Pediatr Obes.

[12] Wesarg C., Van Den Akker A.L., Oei N.Y.L., Hoeve M., Wiers R.W. (2020). Identifying pathways from early adversity to psychopathology: A review on dysregulated HPA axis functioning and impaired self-regulation in early childhood. Eur J Dev Psychol.

[13] Bunea I.M., Szentágotai-Tătar A., Miu A.C. (2017). Early-life adversity and cortisol response to social stress: A meta-analysis. Transl Psychiatry.

[14] Colich N.L., Rosen M.L., Williams E.S., McLaughlin K.A. (2020). Biological aging in childhood and adolescence following experiences of threat and deprivation: A systematic review and meta-analysis. Psychol Bull.

[15] Sumner J.A., Gao X., Gambazza S., Dye C.K., Colich N.L., Baccarelli A.A. (2023). Stressful life events and accelerated biological aging over time in youths. Psychoneuroendocrinology.

[16] Gao X., Geng T., Jiang M., Huang N., Zheng Y., Belsky D.W., Huang T. (2023). Accelerated biological aging and risk of depression and anxiety: Evidence from 424,299 UK Biobank participants. Nat Commun.

[17] Sheridan M.A., McLaughlin K.A. (2014). Dimensions of early experience and neural development: Deprivation and threat. Trends Cogn Sci.

[18] McLaughlin K.A., Sheridan M.A. (2016). Beyond cumulative risk: A dimensional approach to childhood adversity. Curr Dir Psychol Sci.

[19] McLaughlin K.A., Sheridan M.A., Lambert H.K. (2014). Childhood adversity and neural development: Deprivation and threat as distinct dimensions of early experience. Neurosci Biobehav Rev.

[20] Shaul M., Whittle S., Silk T.J., Vijayakumar N. (2024). Pubertal timing mediates the association between threat adversity and psychopathology. Psychol Med.

[21] Rampersaud R., Protsenko E., Yang R., Reus V., Hammamieh R., Wu G.W.Y. (2022). Dimensions of childhood adversity differentially affect biological aging in major depression. Transl Psychiatry.

[22] Achenbach T.M., Rescorla L.A. (2001).

[23] Ribbe D. (1996). Measurement of Stress, Trauma, and Adaptation.

[24] Sanders B., Becker-Lausen E. (1995). The measurement of psychological maltreatment: Early data on the Child Abuse and Trauma Scale. Child Abuse Negl.

[25] Kantor G.K., Holt M.K., Mebert C.J., Straus M.A., Drach K.M., Ricci L.R. (2004). Development and preliminary psychometric properties of the multidimensional neglectful behavior scale-child report. Child Maltreat.

[26] Reitman D., Rhode P.C., Hupp S.D.A., Altobello C. (2002). Development and validation of the parental authority questionnaire—Revised. J Psychopathol Behav Assess.

[27] Hooley J.M., Teasdale J.D. (1989). Predictors of relapse in unipolar depressives: Expressed emotion, marital distress, and perceived criticism. J Abnorm Psychol.

[28] Capaldi D.M., Patterson G.R. (1989).

[29] Barber B.K. (1996). Parental psychological control: Revisiting a neglected construct. Child Dev.

[30] Robinson C.C., Mandleco B., Olsen S.F., Hart C.H. (1995). Authoritative, authoritarian, and permissive parenting practices: Development of a new measure. Psychol Rep.

[31] Robinson C.C., Mandleco B., Olsen S.F., Hart C.H., Touliatos J., Perlmutter B.F., Holden G.W. (2001). Handbook of Family Measurement Techniques.

[32] Achenbach T.M., Cautin R.L., Lilienfeld S.O. (2014). The Encyclopedia of Clinical Psychology.

[33] Zimet G.D., Dahlem N.W., Zimet S.G., Farley G.K. (1988). The Multidimensional Scale of Perceived Social Support. J Pers Assess.

[34] OEHHA (2021). CalEnviroScreen 4.0 [text]. https://oehha.ca.gov/calenviroscreen/report/calenviroscreen-40.

[35] Singh G.K. (2003). Area deprivation and widening inequalities in US mortality, 1969–1998. Am J Public Health.

[36] Kind A.J.H., Jencks S., Brock J., Yu M., Bartels C., Ehlenbach W. (2014). Neighborhood socioeconomic disadvantage and 30-day rehospitalization: A retrospective cohort study. Ann Intern Med.

[37] Ridge N.W., Warren J.S., Burlingame G.M., Wells M.G., Tumblin K.M. (2009). Reliability and validity of the youth outcome questionnaire self-report. J Clin Psychol.

[38] van Buuren S., Groothuis-Oudshoorn K. (2011). mice: Multivariate Imputation by Chained Equations in R. J Stat Soft.

[39] Dray S., Josse J. (2015). Principal component analysis with missing values: A comparative survey of methods. Plant Ecol.

[40] Nassiri V., Lovik A., Molenberghs G., Verbeke G. (2018). On using multiple imputation for exploratory factor analysis of incomplete data. Behav Res Methods.

[41] Antonacci C., Buthmann J.L., Uy J.P., Khosravi P., Lee Y., Gotlib I.H. (2025). Frontolimbic connectivity and threat-related psychopathology: A data-driven test of models of early adversity. Dev Psychobiol.

[42] Broekhof R., Nordahl H.M., Bjørnelv S., Selvik S.G. (2022). Prevalence of adverse childhood experiences and their co-occurrence in a large population of adolescents: A Young HUNT 3 study. Soc Psychiatry Psychiatr Epidemiol.

[43] Liu S., Kuppens P., Bringmann L. (2021). On the use of empirical Bayes estimates as measures of individual traits. Assessment.

[44] Winkler A.M., Renaud O., Smith S.M., Nichols T.E. (2020). Permutation inference for canonical correlation analysis. Neuroimage.

[45] Guidi J., Lucente M., Sonino N., Fava G.A. (2021). Allostatic load and its impact on health: A systematic review. Psychother Psychosom.

[46] McEwen B.S. (1998). Stress, adaptation, and disease. Allostasis and allostatic load. Ann N Y Acad Sci.

[47] Reilly J.J., Kelly J. (2011). Long-term impact of overweight and obesity in childhood and adolescence on morbidity and premature mortality in adulthood: Systematic review. Int J Obes (Lond).

[48] Llewellyn A., Simmonds M., Owen C.G., Woolacott N. (2016). Childhood obesity as a predictor of morbidity in adulthood: A systematic review and meta-analysis. Obes Rev.

[49] Belsky J., Schlomer G.L., Ellis B.J. (2012). Beyond cumulative risk: Distinguishing harshness and unpredictability as determinants of parenting and early life history strategy. Dev Psychol.

[50] Ellis B.J., Boyce W.T., Belsky J., Bakermans-Kranenburg M.J., van Ijzendoorn M.H. (2011). Differential susceptibility to the environment: An evolutionary—Neurodevelopmental theory. Dev Psychopathol.

[51] Ridout K.K., Coe J.L., Parade S.H., Marsit C.J., Kao H.T., Porton B. (2020). Molecular markers of neuroendocrine function and mitochondrial biogenesis associated with early life stress. Psychoneuroendocrinology.

[52] Picard M., McEwen B.S. (2018). Psychological stress and mitochondria: A systematic review. Psychosom Med.

[53] Lee H.C., Wei Y.H. (2005). Mitochondrial biogenesis and mitochondrial DNA maintenance of mammalian cells under oxidative stress. Int J Biochem Cell Biol.

[54] Nelson C.A., Gabard-Durnam L.J. (2020). Early adversity and critical periods: Neurodevelopmental consequences of violating the expectable environment. Trends Neurosci.

[55] Hertzman C., Boyce T. (2010). How experience gets under the skin to create gradients in developmental health. Annu Rev Public Health.

[56] Gluckman P.D., Hanson M.A., Buklijas T. (2010). A conceptual framework for the developmental origins of health and disease. J Dev Orig Health Dis.

[57] Weaver I.C.G., Cervoni N., Champagne F.A., D’Alessio A.C., Sharma S., Seckl J.R. (2004). Epigenetic programming by maternal behavior. Nat Neurosci.

[58] Aubert G., Hills M., Lansdorp P.M. (2012). Telomere length measurement-caveats and a critical assessment of the available technologies and tools. Mutat Res.

[59] Factor-Litvak P., Susser E., Kezios K., McKeague I., Kark J.D., Hoffman M. (2016). Leukocyte telomere length in newborns: Implications for the role of telomeres in human disease. Pediatrics.

[60] Miller G.E., Chen E., Fok A.K., Walker H., Lim A., Nicholls E.F. (2009). Low early-life social class leaves a biological residue manifested by decreased glucocorticoid and increased proinflammatory signaling. Proc Natl Acad Sci U S A.

[61] Caspi A., Houts R.M., Belsky D.W., Goldman-Mellor S.J., Harrington H., Israel S. (2014). The p factor: One general psychopathology factor in the structure of psychiatric disorders?. Clin Psychol Sci.

[62] Lahey B.B., Krueger R.F., Rathouz P.J., Waldman I.D., Zald D.H. (2017). A hierarchical causal taxonomy of psychopathology across the life span. Psychol Bull.

[63] Ullsperger J.M., Nikolas M.A. (2017). A meta-analytic review of the association between pubertal timing and psychopathology in adolescence: Are there sex differences in risk?. Psychol Bull.

[64] Ge X., Natsuaki M.N. (2009). In search of explanations for early pubertal timing effects on developmental psychopathology. Curr Dir Psychol Sci.

