# Supplementary material for: Early Adversity Imprints Multisystem Biological Aging: A Longitudinal Study of Adversity Dimensions and Aging Phenotypes Across Adolescence
Source: Biol Psychiatry Glob Open Sci. 2026 Jun 24;6(5):100782. doi: 10.1016/j.bpsgos.2026.100782 (PMC13475218; doi:10.1016/j.bpsgos.2026.100782)
Supplement: Supplementary file 1 [file mmc1.pdf]

SUPPLEMENTARY INFORMATION

Early Adversity Imprints Multisystem Biological Aging: A Longitudinal Study of Adversity Dimensions and Aging Phenotypes Across Adolescence

Antonacci *et al.*

1. Table S1: Coding of Demographic Variables
2. Measures of Early Life Adversity/Stress
3. Measures of Biological Aging
4. Figure S1: Principal Components Analysis of Stress Measures
5. CCA with Theory-Driven Adversity Composites
    a. Table S2: Theory-Driven CCA: Results Summary
    b. Figure S2: Theory-Driven CV2 (Threat) Canonical Loadings
6. Sex-Stratified Analyses
7. Figure S3: Participant Flow Diagram
8. Table S3: Biomarker Data Availability by Wave
9. Figure S4: Permutation-Based Null Distributions for Canonical Correlation Analysis
10. Structural Imaging Sensitivity Analysis: Cortical Thickness and Surface Area
    a. Figure S5: Structural Imaging Sensitivity Analysis

1. Table S1: Coding of Demographic Variables

Variable	Coding Scheme/Description
Sex	1 = Male; 2 = Female
Race	1 = White;<br>2 = Black;<br>3 = Hispanic;<br>4 = Asian;<br>5 = Biracial;<br>6 = Other
Income-to-Needs Ratio	To operationalize SES, we calculated the Income-to-Needs ratio for each child's family. The parent who accompanied the child to the laboratory session reported total family income over the previous 12 months and the number of people in their family. We collected income data in bins, with parents reporting income on a 10-point scale as follows: <≤$5000 (*N* = 1); $5001–$10,000 (*N* = 2); $10,001–$15,000 (*N* = 1); $15,001–$25,000 (*N* = 6); $25,001–$35,000 (*N* = 2); $35,001–$50,000 (*N* = 10); $50,001–$75,000 (*N* = 13); $75,000–$100,000 (*N* = 17); $100,001–$150,000 (*N* = 40); ≥$150,000 (*N* = 54). To calculate the INR, we divided the midpoint of the endorsed income bin by the low-income limit for Santa Clara county (80% of the median income) determined by the Department of Housing and Urban Development based on the number of people in the household (https://www.huduser.gov/portal/datasets/il/il2017/2017summary.odn; $84,750 for a family of four within the study period).
Parent Education	1 = less than high school diploma;<br>2 = GED/high school diploma;<br>3 = some college;<br>4 = two-year college degree;<br>5 = four-year college degree;<br>6 = master's degree;<br>7 = professional degree;<br>8 = doctorate

2. Measures of Early Life Adversity/Stress

We assessed exposure to early life adversity using 16 measures capturing diverse domains of stress exposure. Composite scores from the following measures were submitted to principal components analysis (PCA) for dimensionality reduction.

Traumatic Events Screening Inventory. The Traumatic Events Screening Inventory for Children (TESI) is a structured interview for systematically assessing exposure to trauma across 33 domains, including accidents, illness, neglect, abuse, and domestic violence (1). The TESI was administered at the baseline assessment and each item endorsed by participants was immediately followed by probes from the interviewer to determine risk, if any, to the participant's safety and well-being. Imminent risks were reported to the principal investigator, who undertook safeguarding procedures as outlined in the study's ethics approvals. During the interview, participants retrospectively reported their exposure to stressors and their own subjective rating of severity for each endorsed event. Following the interview, an independent panel, masked to the participant's identity and subjective TESI ratings, assigned an objective severity rating for each reported event in accordance with the UCLA Life Stress Interview Rating manual (2,3; ICC=0.99). Ratings ranged from 0 (not impactful) to 4 (extremely severe impact), in half point increments. Objective stress severity was defined as the sum of the maximum panel-rated severity scores for each type of event reported. Subjective stress severity was defined as the sum of the maximum participant-rated severity scores for each type of event endorsed.

Ratings on the TESI were also summed to yield 'Threat' and 'Unpredictability' scores, which were derived from prior work (4,5). Items in the Threat subscale included the following: family verbal conflict (including verbal threats to the participant and/or family members), bullying involving threat or experience of physical victimization, community violence, community instability (including active shooter or bomb threats and hearing gunshots either at home or school), domestic violence, community verbal conflict, emotional abuse, physical abuse,

mugging/robbery, war/terrorism, sexual abuse, threats of domestic violence, kidnapping, threats of physical abuse, and witnessing sexual abuse. The Unpredictability subscale included the following items: moved households, death of someone close, parental divorce, separation from family related to legal issues or travel, family legal problems, experience a disaster, witness to illness/injury, experience illness/injury, witness to accident, experience accident, and family financial insecurity.

Parenting. Several measures of parenting quality were administered to participants at the baseline assessment, including the Multidimensional Neglectful Behavior Scale (MNBS; 6) total score), the Parental Authority Questionnaire (PAQ; 7; authoritative parenting subscale), the Perceived Criticism Scale (PCS; 8; parental criticism subscale), the Parental Monitoring Scale (PMS; 9; total score, reversed), the Parental Psychological Control Scale (PPC; 10,11; control subscale); and the Parenting Styles and Dimensions Questionnaire (PSDQ; 12; warmth questionnaire, reversed). The parenting measures were scored such that higher values corresponded to 'harsher' parenting behaviors.

Parent Psychopathology. Parents also completed the Adult Self Report (13), a 126-item inventory of behavioral problems and adaptive functioning. The self-report questionnaire assesses participants across the following syndrome scales: anxious/depressed, withdrawn, somatic complaints, thought problems, attention problems, aggressive behavior, rule-breaking behavior, and intrusive behavior. Items are scored on a 3-point Likert scale (0: "Not True" to 2: "Very True or Often True"). The eight syndrome scales are then combined to produce a Total Problems raw score, which was used as a measure of stress in the current study.

CalEnviroScreen. The California Communities Environmental Health Screening Tool (CalEnviroScreen 4.0; 14) is a multifactored geographic database assessing socioeconomic, environmental, and public health resource quality across the state of California at the census tract level. CalEnviroScreen consists of 21 variables that are combined to yield two subscales: 'Pollution Burden' and 'Population Characteristics.' The Pollution Burden subscale includes

neighborhood ozone levels, fine particulate matter (PM2.5), lead and diesel exposure, drinking water contaminants, and proximity to hazardous waste facilities, among other indicators. The Population Characteristics subscale includes cardiovascular disease rate, neighborhood education and poverty levels, unemployment rate, and housing burden. The CalEnviroScreen 4.0 uses census tract boundaries from the 2010 census; each of the indicators comprising the Pollution Burden and Population Characteristics subscales are sourced from data repositories with collection dates ranging from 2009-2019. Scores on the CalEnviroScreen subscales were calculated based on participants' residential address at the baseline assessment (date range=2013-2017) and were scored such that higher values indicated higher pollution burden and greater neighborhood and socioeconomic disadvantage.

Multidimensional Scale of Perceived Social Support. The Multidimensional Scale of Perceived Social Support (MSPSS; 15) is a 12-item self-report instrument designed to assess perceived adequacy of social support from family, friends, and the participant's significant other (if applicable). Participants responded to each item on a 7-point Likert scale (1: "Very Strongly Disagree" to 7: "Very Strongly Agree"). Total scores ranged from 12-84 and were reverse-scored such that higher scores indicated lower perceived social support. The MSPSS has been found to have satisfactory internal consistency (α=0.91) and re-test reliability (ICC=0.84; 16).

Early Life Stress Questionnaire. The Early Life Stress Questionnaire (ELSQ; 17) is a brief self-report measure assessing retrospective exposure to 19 categories of stressful or adverse events occurring prior to age 17, including physical, emotional, and sexual abuse; neglect; parental divorce or separation; domestic violence; family financial hardship; and loss of a close family member, among others. Participants indicated via binary response (yes/no) whether they had experienced each event during childhood. Total scores represent a simple count of endorsed adversity categories and range from 0 to 19, with higher scores reflecting greater cumulative early adversity exposure. The ELSQ was administered at the baseline assessment.

Area Deprivation Index. The Area Deprivation Index (ADI; 18,19) is a composite measure of neighborhood-level socioeconomic disadvantage derived from U.S. Census and American Community Survey data at the census block group level. The ADI aggregates 17 indicators across four domains – income and employment, education, housing quality, and household characteristics – into a single deprivation score. Participants' residential addresses at the baseline assessment were geocoded and linked to their corresponding census block group to obtain ADI scores, which are expressed as national percentile rankings (ADI-NATRANK) ranging from 1 (least deprived) to 100 (most deprived). National rankings were used in the present study to permit comparisons across geographically diverse participants. Scores were oriented such that higher values indicated greater neighborhood socioeconomic disadvantage.

3. Measures of Biological Aging

Body Mass Index. Height and weight were measured at each assessment wave by trained research staff using a stadiometer and calibrated digital scale, respectively. Body mass index (BMI) was calculated using the standard formula: weight (kg) divided by height in meters squared (kg/m^2).

Mitochondrial DNA Copy Number (mtDNA). mtDNA was previously assayed and analyzed in the current sample as described in (20). Detection of a 69 bp fragment of the ND1gene in mtDNA (nucleotides 3485-3553) and an 87 bp fragment of RNase P (TaqMan® Copy Number Reference Assay, human, RNase P, cat# 4403328, Life Technologies) by TaqMan assays was used to determine the relative copy number of mtDNA per diploid nuclear genome. The primer and probe sequences for ND1 were:

- ND1-forward 5'-CCCTAAAACCCGCCACATCT-3'
- ND1-reverse 5'-GAGCGATGGTGAGAGCTAAGGT-3'
- ND1-FAM probe 5' FAM-CCATCACCCTCTACATCACCGCCC-TAMRA-3'

All primers were ordered from IDT. RNase P probe was VIC-labelled. The reaction contained 12.5 ng of genomic DNA, 100nM of ND1 probe, 300 nM of ND1-forward primer and ND1-reverse primer each, 1X RNase P copy number Reference Assay, 1X LightCycler® 480 Probe Master (Roche, cat# 04902343001) in a 10 ul reaction. All samples were run in triplicate wells in 384-well plates in a Roche Lightcyler 480. PCR condition was PCR cycle: 95°C 10 min 45 cycles of 95 °C 10 sec, 60°C 30 sec, 72°C 1 sec with data acquisition at 72°C. Crossing point (CP) for each well was derived by the LightCycler 480 program using the second derivation method. Relative copy number was calculated by the following formula:

- Relative mtDNA copy number/diploid genome=$2^{(CP\_ND1 - CP\_RNaseP)} \times 2$.

mtDNA was assayed in two batches. Formal inter- and intra-assay CVs were not collected prospectively; however, QC samples run during assay setup demonstrated inter-assay CV=3.4% and intra-assay CV=1.7%.

Salivary Cortisol. We used a diurnal cortisol collection protocol adopted from (21,22). At each assessment, participants were given instructions for the salivary cortisol sample collection, both verbally and in writing. Participants then collected salivary samples at home for two consecutive weekdays with SalivaBio Children's Swabs (Salimetrics, LLC). Samples were to be taken at the following times: 1) at waking while still in bed; 2) 30 minutes after waking; 3) mid-afternoon at 3:00pm; and 4) in the evening two hours after dinner. Participants recorded the specific time of sample collection on a collection sheet. They stored the samples in home freezers before returning them to the lab, where they were stored in a -20°C freezer in the Stanford University Psychology Department. When enough samples were collected, they were assayed at Salimetrics in accordance with the company's protocol, described in the following boilerplate:

"Prior to testing, samples were stored at -80°C before being shipped on dry ice to the Salimetrics SalivaLab (Carlsbad, CA). Samples were thawed to room temperature, vortexed, and then centrifuged for 15 minutes at approximately 3,500 RPM (1,500 x g) immediately before performing the assay. Samples were tested for salivary cortisol using a high sensitivity enzyme immunoassay (Cat. No. 1-3002). Sample test volume was 25 μl of saliva per determination. The assay has a lower limit of sensitivity of 0.007 μg/dL, a standard curve range from 0.012-3.0 μg/dL, and an average intra-assay coefficient of variation of 4.60%, and an average inter-assay coefficient of variation of 6.00%, which meets the manufacturers' criteria for accuracy and repeatability in Salivary Bioscience, and exceeds the applicable NIH guidelines for Enhancing Reproducibility through Rigor and Transparency."

Finally, as described in (23), we computed area under the curve with respect to ground (AUCg), indexing total cortisol produced throughout the day (24). Outlying values were winsorized using a 5% threshold.

Telomere Length. Telomere length in this sample was quantified as previously described in (20) and (25), and the assay was adapted from the method originally published by (26). Briefly, genomic DNA was purified from 500 μL of saliva collected in the Oragene DNA Kit (DNA Genotek, Kanata, ON, Canada) with the DNA Agencourt DNAdvance Kit (cat. No. A48705; Beckman Coulter Genomics, Brea, CA, USA). DNA was quantified by Quant-iT PicoGreen dsDNA Assay Kit (cat. No. P7589; Life Technologies, Grand Island, NY, USA) and run on 0.8% agarose gels to check the integrity. DNA samples were stored at -80°C. The cycling profile for T (telomeric) PFC consisted of: denature of 96 °C for 1 min; denature at 96 °C for 1 s, anneal/extend at 54 °C for 60 s, with fluorescence data collection, 30 cycles. The cycling profile for S (single-copy gene) PCR consisted of: denature at 96 °C for 1 min, denature at 95 °C for 1 s, anneal at 58 °C for 1 s, extend at 72 °C for 20 s, hold at 83 °C for 5 s with data collection, 35 cycles.

The primers for the telomere PCR were tel1b (5'-CGGTTT(GTTTGG)5GTT-3'), used at a final concentration of 100 nM, and tel2b (5'-GGCTTG(CCTTAC)5CCT-3'), used at a final concentration of 900 nM. The primers for the single-copy gene (human β-globin) PCR were hbg1 (5'-GCTTCTGACACAACTGTGTTCCTAGC-3'), used at a final concentration of 300 nM, and hbg2 (5'-CACCAACTTCATCCACGTTCACC-3'), used at a final concentration of 700 nM. The final reaction mix contained 20 mM Tris-HC1 (pH 8.4), 50 mM KC1, 200 μM each dNTP, 1% DMSO, 0.4 x Syber Green I, 22 ng Escherichia coli DNA per reaction, 0.4 U of Platinum Taq DNA polymerase (Life Technologies, Carlsbad, CA, USA) per 11 μl reaction and 7 ng of genomic DNA. Tubes containing 26, 8.75, 2.9, 0.97, 0.324 and 0.108 ng of a reference DNA (pooled human genomic DNA from buffy coat, Roche Diagnostics Cat# 1691112001) were included in each PCR run so that the quantity of targeted templates in each research sample

could be determined relative to the reference DNA sample by the standard curve method. The same reference DNA was used for all PCR runs.

Telomere length was assayed in three batches. To control for interassay variability, eight control DNA samples were included in each run. In each batch, the T/S ratio of each control DNA was divided by the average T/S for the same DNA from 10 runs to get a normalizing factor. This was carried out for all eight samples and the average normalizing factor for all eight samples was used to correct the participant DNA samples to get the final T/S ratio. The T/S ratio for each sample was measured two times. When the duplicate T/S value and the initial value varied by more than 7%, the sample was run a third time and the two closest values were reported. Using this method, the inter-assay CVs were 2.3% for the first batch, 2.1% for the second batch, and 2.2% for the third batch. Intra-assay CVs were not collected for individual batches.

Brain Age Gap

MRI Acquisition and Structural Processing. Neuroimaging data were collected at the Center for Cognitive and Neurobiological Imaging at Stanford University using a 3T General Electric Discovery MR750. T1-weighted (T1w) structural images were acquired at each assessment wave using a sagittal spoiled gradient echo sequence: echo time (TE)=2.4 ms, repetition time (TR)=6.3 ms, isometric voxel size = 0.90 mm^3, 186 slices, field of view (FOV)=230 mm, flip angle (FA)=12°. Raw images were manually inspected for quality and gross artifacts prior to analysis. T1w images were processed using FreeSurfer's recon-all pipeline (v6.0.1; surfer.nmr.mgh.harvard.edu), which performs skull stripping, subcortical segmentation, and cortical surface reconstruction. From each processed image, we extracted 189 structural features for use as brain age model inputs: 53 global and subcortical volumetric measures from the aseg segmentation, and bilateral cortical surface area and gray matter volume for 34 regions per hemisphere from the Desikan-Killiany atlas (aparc parcellation), using the *asegstats2table* and *aparcstats2table* utilities.

Brain Age Estimation. Predicted brain age was estimated using the Developmental Brain Age model (27), an XGBoost algorithm trained specifically on structural MRI data from 1,299 typically developing adolescents aged 9-19 years (*M*=13.5, *SD*=3.04) and validated in an independent holdout sample of 322 adolescents. The model accepts the 189 FreeSurfer-derived structural features described above and outputs a predicted brain age in years. The pre-trained model weights are publicly available (github.com/GitDro/DevelopmentalBrainAge). Brain age gap (BAG) at each wave was computed as predicted brain age minus chronological age at scan, such that positive values indicate neurobiological advancement relative to age-matched peers.

Age Bias Correction. A well-documented artifact of machine learning brain age models is regression to the mean, whereby younger individuals tend to be over-estimated and older individuals under-estimated, producing a spurious negative correlation between BAG and chronological age. Consistent with standard practice (28), we corrected for this bias at each assessment wave by regressing BAG on chronological age and retaining the unstandardized residuals (BrainAgeGap_Resid) as the corrected brain age measure for all analyses. Prior to correction, BAG was significantly correlated with scan age across waves (r=-0.45 to -0.68), confirming the presence of age-related bias; after correction, these correlations were reduced to approximately zero by construction.

<u>Pubertal Stage.</u> Pubertal development was assessed at each wave via participant self-report using illustrated Tanner staging drawings (29). Participants viewed sex-specific schematic illustrations depicting the five stages of pubertal maturation — ranging from Stage 1 (prepubertal) to Stage 5 (fully mature) — and selected the image that best matched their current physical development. For female participants, ratings were based on breast development and pubic hair; for male participants, ratings were based on genital development and pubic hair. Self-reported Tanner staging using illustrated drawings has demonstrated adequate validity

relative to physician assessment, with correlations between self- and clinician-rated stages typically ranging from r=.60 to .80 across physical characteristics (30,31).

4. Figure S1: Principal Components Analysis of Stress Measures

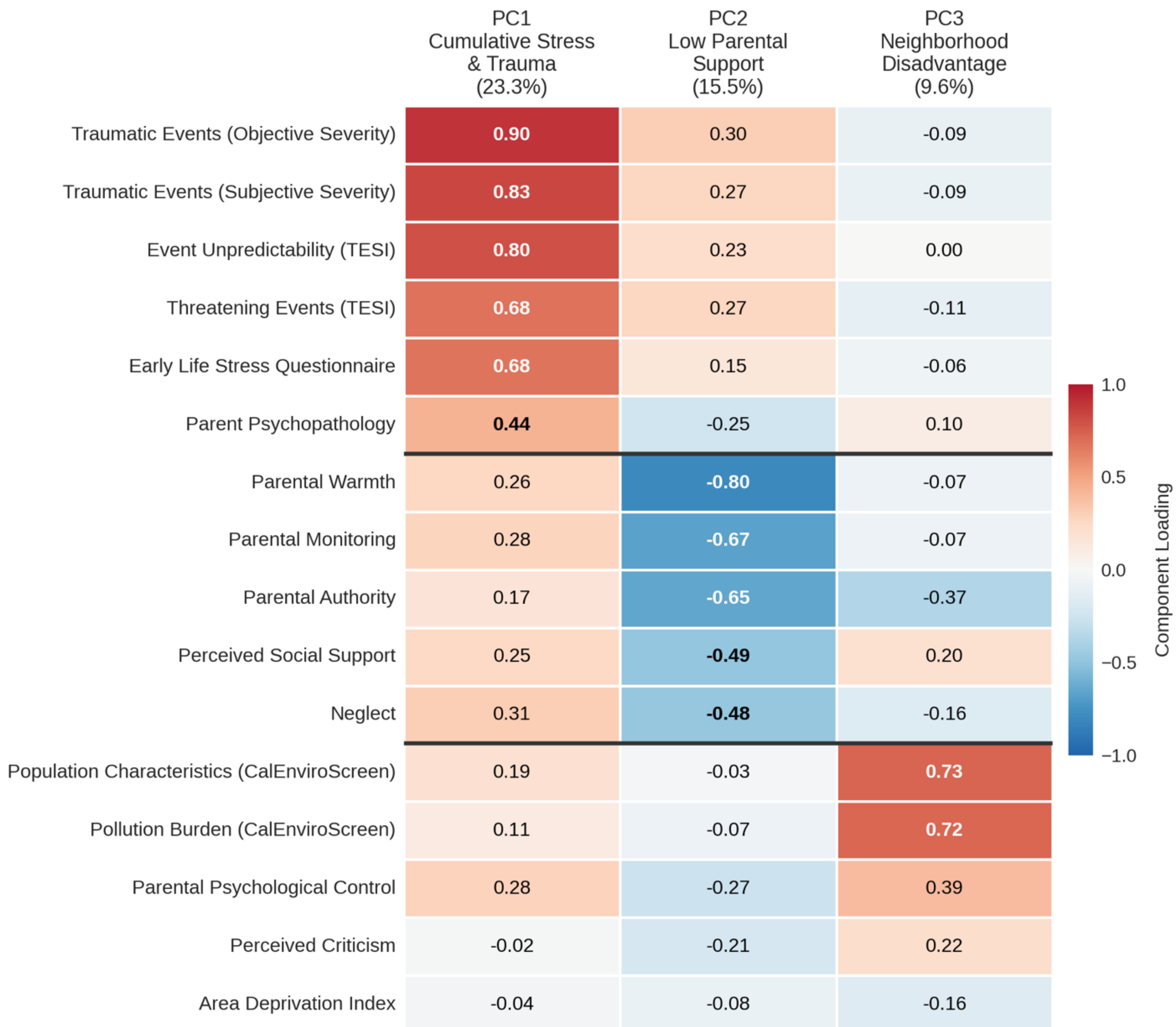

Note. Varimax-rotated PCA loading for stress exposure variables. Heatmap displays factor loadings for each of the 16 stress exposure variables across three retained principal components (PCs), determined via parallel analysis (1,000 permutation samples; 95th percentile criterion). Missing data were handled via multiple imputation by chained equations (MICE; *m*=10, predictive mean matching) prior to PCA. Components are labeled according to their dominant loading pattern: PC1 (*Cumulative Stress & Trauma*; 23.3% variance explained) loaded most strongly on measures of traumatic event exposure; PC2 (*Low Parental Support*; 15.5%) loaded negatively on parenting quality, warmth, and monitoring; and PC3 (*Neighborhood Disadvantage*; 9.6%) loaded most strongly on environmental burden indices. Cell values reflect pattern matrix loadings; bolded values indicate loadings ≥ |.40|.

5. CCA with Theory-Driven Adversity Composites

Supplemental Methods: Theory Driven Adversity Composites

To complement the data-driven PCA approach and provide a more direct test of the DMAP framework, we constructed theory-driven adversity composites by categorizing the 16 stress measures *a priori* into threat and deprivation dimensions based on McLaughlin and Sheridan's published definitions (32,33). Threat was indexed by measures of direct harm, interpersonal violence, and environmental unpredictability: TESI-C objective and subjective sum severity, threat subscale, and unpredictability subscale; ELSQ total score; parental criticism (PCS); parental psychological control (PPC); and parent psychopathology (ASR Total Problems, included under threat based on the formulation that parental psychiatric symptoms introduce environmental unpredictability). Deprivation was indexed by measures reflecting absence of expected environmental inputs and resources: neglect (MNBS), parenting authority (PAQ), low monitoring (PMC), low warmth (PSDQ warmth), low perceived social support (MSPSS), CalEnviroScreen pollution burden score, CalEnviroScreen population characteristics score, and Area Deprivation Index. Within each category, variables were *z*-scored and averaged to form a single composite. The resulting threat and deprivation composites were modestly correlated (r=.195). CCA was then conducted using the two composites as the predictor matrix and the same six baseline biomarkers as in the primary analysis, following identical procedures (SVD-based CCA; 5,000 permutations; seed=42). Slopes and psychopathology prediction analyses were conducted as described in the main text.

Supplemental Results: Theory-Driven CCA

CCA using the theory-driven composites identified one significant canonical variate. CV2, dominated by the threat composite (loading = +0.96; deprivation loading = -0.07), was significant (r=0.209, p=.003) and indexed a biomarker profile characterized by elevated BMI (+0.93), older brain age gap (+0.51), and earlier pubertal stage (+0.38), broadly consistent with

the threat phenotype identified in the primary analysis (see Supplemental Table 2 and Supplemental Figure 2). CV1, dominated by the deprivation composite (loading = +0.97), was not statistically significant (r=0.245, p=.146). The non-significance of the deprivation CV may reflect reduced sensitivity relative to the data-driven approach; in the primary analysis, deprivation decomposed into two orthogonal PCA components (emotional/relational and material/structural) entered as separate predictors, allowing the CCA to weight each according to its differential biomarker relevance. Averaging all deprivation items with equal weights constrains this optimization and may dilute the signal from more informative items. As in the primary analysis, neither canonical variate reached significance in the slopes CCA (CV1: p=.078; CV2: p=.757), consistent with a developmental imprinting model in which early adversity shapes biological setpoints rather than driving continued dysregulation across adolescence.

Supplemental Results: Theory-Driven Threat Profile and Later Psychopathology

We next examined whether the theory-driven CV2 scores predicted psychopathology six years later, restricting analyses to the threat profile (CV2) given that the deprivation profile (CV1) did not reach significance. Replicating the primary analysis, the threat stress composite score significantly predicted YSR Total Problems (β=8.37, SE = 2.36, 95% CI [3.71, 13.03], p<.001), Internalizing Problems (β=2.39, SE=0.90, 95% CI [0.61, 4.17], p=.009), and Externalizing Problems (β=2.41, SE=0.69, 95% CI [1.05, 3.78], p<.001) at T4, with no significant stress-by-biomarker interactions (all ps>.50). The threat biomarker composite score showed modest but significant independent associations with Total (β=4.83, SE=1.99, 95% CI [0.88, 8.77], p=.017) and Externalizing Problems in this model (β=1.19, SE=0.58, 95% CI [0.04, 2.35], p=.043), but not Internalizing (β=1.36, SE=0.76, 95% CI [-0.14, 2.86], p=.075). As noted in the main text, the analogous effect was not significant in the primary analysis, an effect likely due to strong stress-biomarker coupling inherent to CCA. The somewhat lower canonical correlation in the theory-driven model (r=.209 vs. r=.244) suggests reduced coupling, which may afford the biomarker score greater unique predictive contribution here.

Table S2: Theory-Driven CCA: Results Summary

(A) Canonical Correlations

Model	Canonical Variate	*r*	*p*
Intercepts Model (Baseline Biomarkers)			
	CV1 (Deprivation)	.245	.146
	CV2 (Threat)	.209	**.003****
Slopes Model (Biomarker Trajectories)			
	CV1	.263	.078
	CV2	.084	.757

Note. *p*-values from permutation test (5,000 permutations). ** indicates $p < .01$.

(B) Canonical Loadings: Intercepts Model

Variable	**CV1 Loadings** (Deprivation)	**CV2 Loadings** (Threat)
Stress Composites		
Threat Composite	+0.43	+0.96
Deprivation Composite	+0.97	-0.07
Biomarker Intercepts		
mtDNA Copy Number	+0.73	-0.21
Telomere Length	+0.44	-0.06
Cortisol AUC	+0.37	-0.05
BMI	+0.00	+0.93
Pubertal Stage	+0.64	+0.38
Brain Age Gap	-0.17	+0.51

Note. Loadings are Pearson correlations between original variables and canonical variate scores. Only the intercepts model is shown; the slopes model yielded no significant variates.

(C) Theory-Driven CV2 (Threat) and T4 Psychopathology (N = 142)

Outcome	Term	*β*	*SE*	95% CI	*p*	R^2
**YSR Total Problems**	Stress CV2	+8.37	2.36	[3.71, 13.03]	**<.001**	.151
	Bio CV2	+4.83	1.99	[0.88, 8.77]	**.017**	-
	Stress x Bio	-1.10	1.67	[-4.41, 2.20]	.510	-
**YSR Internalizing**	Stress CV2	+2.39	0.90	[0.61, 4.17]	**.009**	.131
	Bio CV2	+1.36	0.76	[-0.14, 2.86]	.075	-
	Stress x Bio	-0.37	0.64	[-1.63, 0.89]	.562	-
**YSR Externalizing**	Stress CV2	+2.41	0.69	[1.05, 3.78]	**<.001**	.128

Outcome	Term	β	SE	95% CI	p	R^2
	Bio CV2	+1.19	0.58	[0.04, 2.35]	**.043**	-
	Stress x Bio	-0.27	0.49	[-1.24, 0.70]	.580	-

Note. All models include age and sex as covariates. R^2 reported for the Stress CV2 term row only (model-level). CI = 95% confidence interval.

Figure S2: Theory-Driven CV2 (Threat) Canonical Loadings

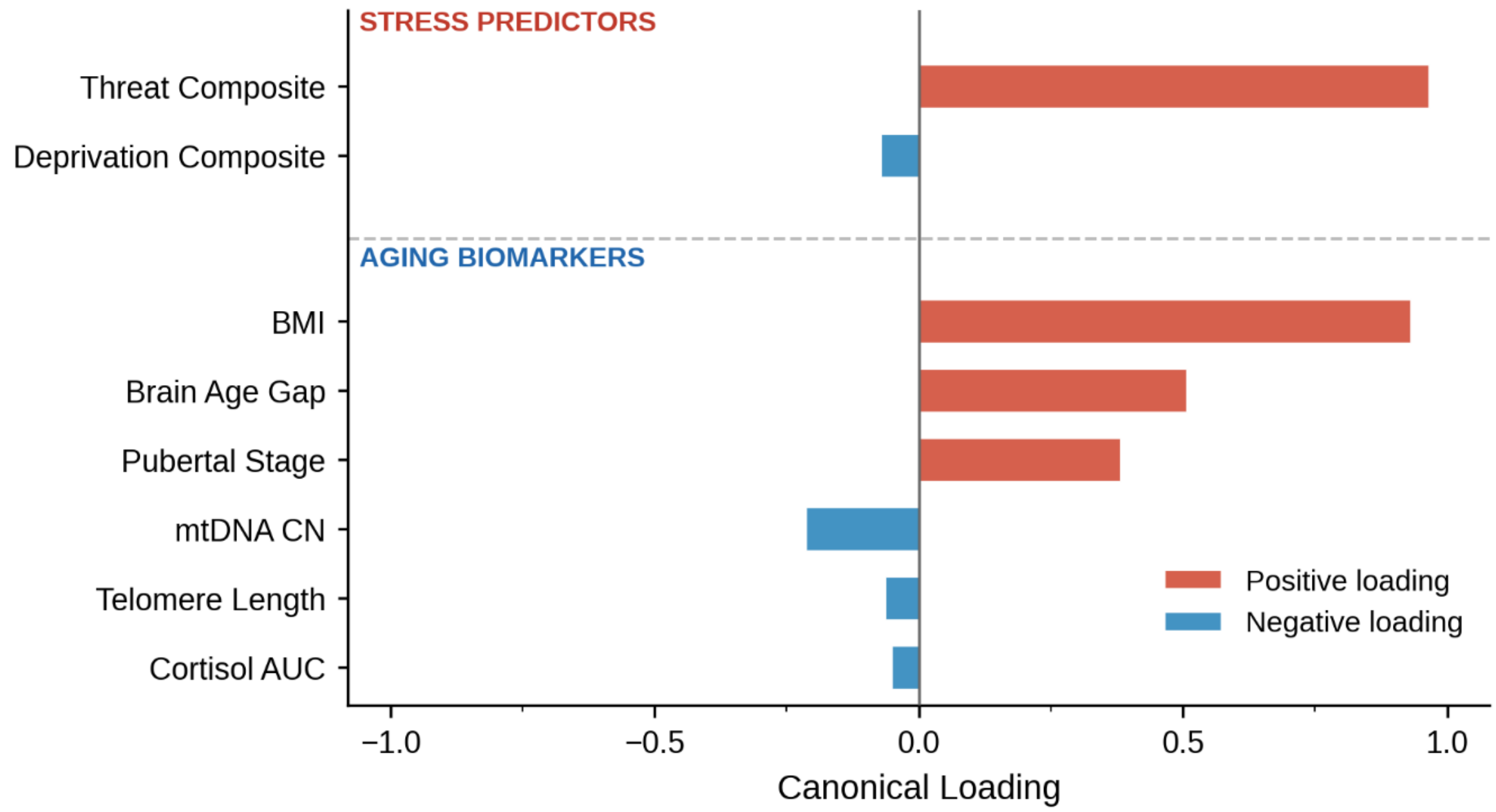

Note. Theory-driven threat Profile (CV2) canonical loadings. Bars represent Pearson correlations between each variable and the CV2 canonical variate score, derived from CCA using *a priori* threat and deprivation composites. Variables are sorted within section by absolute loading magnitude. Red = positive loading; blue = negative loading. Only the threat profile (CV2), but not the deprivation profile (CV1) was significant (r=0.209, p=.003; permutation testing, 5,000 permutations).

6. Sex-Stratified Analyses

Supplemental Methods: Sex-Stratified Analyses

To examine possible sex differences in stress-biomarker coupling, we repeated the primary intercepts CCA separately for females (*n*=132) and males (*n*=93), following identical procedures (*z*-scored predictors and outcomes, 5,000 permutation tests, seed=42). We also ran slopes models for each sex. All analyses used the same three data-driven stress PCs and six biomarker intercepts (or slopes) as the primary analysis.

Supplemental Results: Sex-Stratified Analyses

Females. In females, a single canonical variate reached significance (CV1: *r*=0.581, *p*<.001); CV2 and CV3 were non-significant (*p*s=.232 and .130, respectively). The female CV1 stress loadings were distributed broadly across all three stress dimensions (PC2: +0.674; PC1: +0.596; PC3: +0.460), in contrast to the primary analysis in the full sample where CV1 was predominantly deprivation-driven and CV2 was predominantly threat-driven. The biomarker side of the female CV1 was anchored by greater BMI (+0.813), larger brain age gap (+0.740), and more advanced pubertal stage (+0.310), with lower mtDNA copy number (-0.185), shorter telomere length (-0.151), and lower cortisol (-0.213). The failure to recover the deprivation/threat distinction in females may reflect reduced statistical power within the sex-stratified subsample to separate two closely-related adversity dimensions that are resolved more cleanly in the full sample. As with the full samples, the female-only slopes model yielded no significant variates (*p*s>.063).

We next tested whether the “omnibus stress-biomarker” female CV1 canonical variate scores predicted T4 psychopathology approximately six years later, using the same regression framework as in the primary analysis (outcome ~ Stress CV1 + Bio CV1 + Stress x Bio + age; n=86 with complete T4 YSR data). The stress component of CV1 significantly predicted overall YSR Total Problems (β=6.91, *SE*=3.21, 95% CI [0.54, 13.29], *p*=.034; R^2=.197), consistent with

the primary analysis finding that stress canonical scores prospectively predict psychopathology. The biomarker component and stress-by-biomarker interaction did not reach significance for any outcome (*p*s>.236). Effects on YSR Internalizing and Externalizing Problems were in the expected direction but did not reach conventional significance thresholds (*p*s=.058 and .094, respectively), likely reflecting reduced power given the smaller female subsample with complete follow-up data.

Males. No canonical variates reached significance in the male intercepts model (CV1: *r*=0.455, *p*=.065; CV2: *r*=0.311, *p*=.061; CV3: *r*=0.186, *p*=.102). Notably, the two largest canonical correlations both approached but did not cross conventional significance thresholds, suggesting that there may be a stress-biomarker association of comparable magnitude in males but that with the present sample size (n=93) we did not have sufficient power to detect it, particularly given the documented power requirements of CCA (34,35). In the slopes model, CV3 reached significance (r=0.211, p=.042), characterized by neighborhood disadvantage as the dominant stress loading (PC3: +0.914) and shortened telomere length trajectories as the dominant biomarker loading (-0.815). This pattern suggests that neighborhood-level disadvantage is specifically linked to accelerated telomere attrition in males over time; however, given the small sample size and the fact that this was the third canonical variate, this finding should be interpreted with caution and requires replication in a larger sample before firm conclusions can be drawn.

7. Figure S3: Participant Flow Diagram

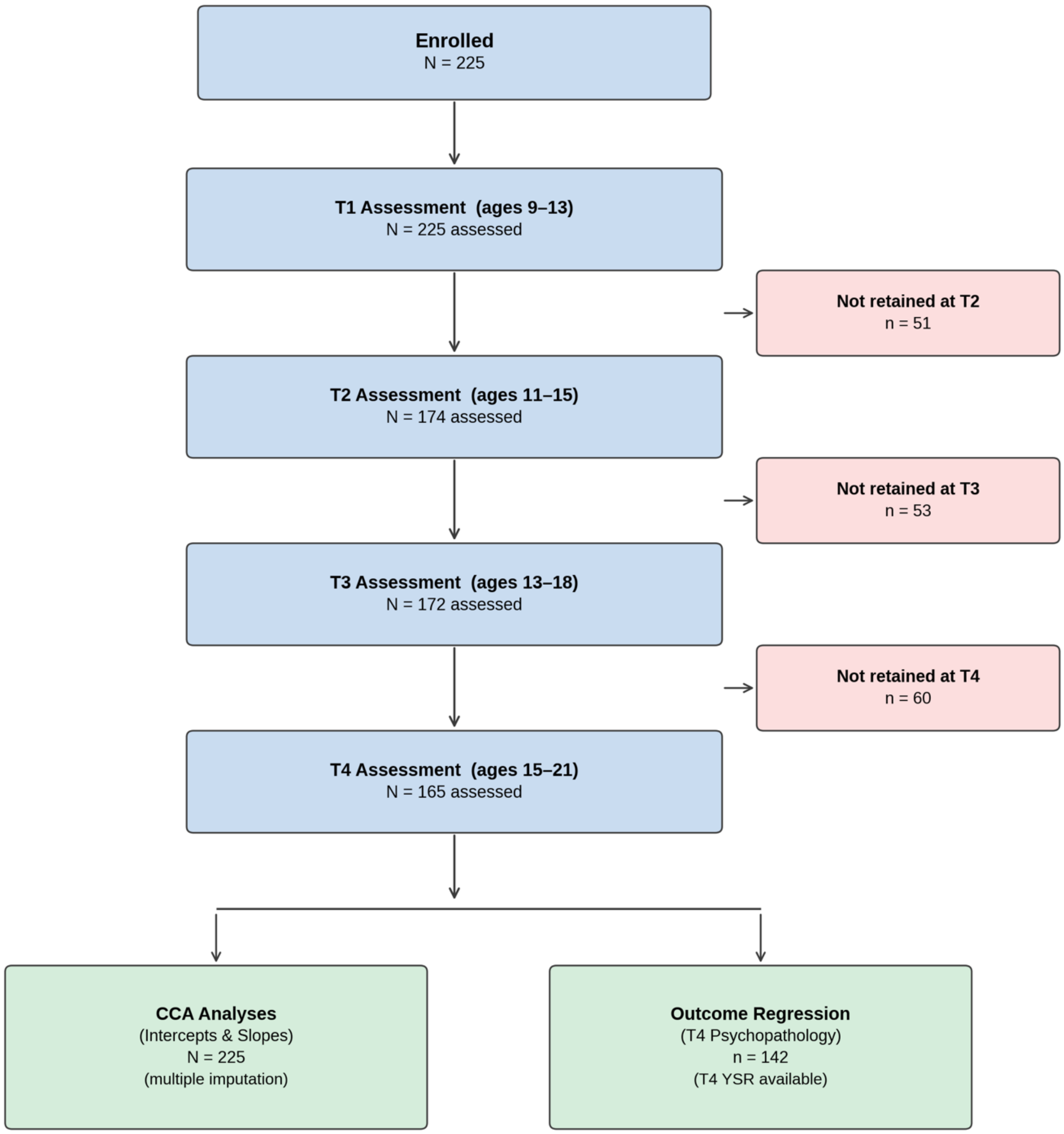

Note. Participant Flow Diagram. Flow of participants across the four assessment waves and into primary analyses. All 225 enrolled participants contributed T1 data. "Not retained" at each wave reflects participants without any biomarker data at that wave; no replacement participants were enrolled across the study period. Within-individual missingness varied by biomarker and wave due to differential assay availability and acquisition logistics (see Supplemental Table S3 for biomarker-specific Ns). Primary CCA analyses included all 225 participants using multiple imputation; outcome regression analyses were restricted to the n=142 participants with T4 YSR data available.

8. Table S3: Biomarker Data Availability by Wave

Biomarker	T1 (ages 9-13)	T2 (ages 11-15)	T3 (ages 13-18)	T4 (ages 15-21)
mtDNA Copy Number	212 (94%)	145 (64%)	142 (63%)	53 (24%)
Telomere Length	212 (94%)	145 (64%)	141 (63%)	51 (23%)
Cortisol AUC	168 (75%)	135 (60%)	116 (52%)	71 (32%)
BMI	205 (91%)	144 (64%)	146 (65%)	113 (50%)
Brain Age Gap	190 (84%)	144 (64%)	124 (55%)	124 (55%)
Pubertal Stage	224 (99%)	164 (73%)	168 (75%)	131 (58%)

Note. Biomarker Data Availability by Assessment Wave. Number and percentage of the analytic sample (N=225) with non-missing data for each biomarker at each assessment wave. The same 225 individuals were followed longitudinally across all four waves; within-individual missingness reflects differential assay availability and acquisition across waves rather than participant dropout. Primary CCA analyses used multiple imputation (MICE) across all 225 participants; outcome regression analyses used the n=142 participants with complete T4 YSR data.

9. Figure S4: Permutation-Based Null Distributions for Canonical Correlation Analysis

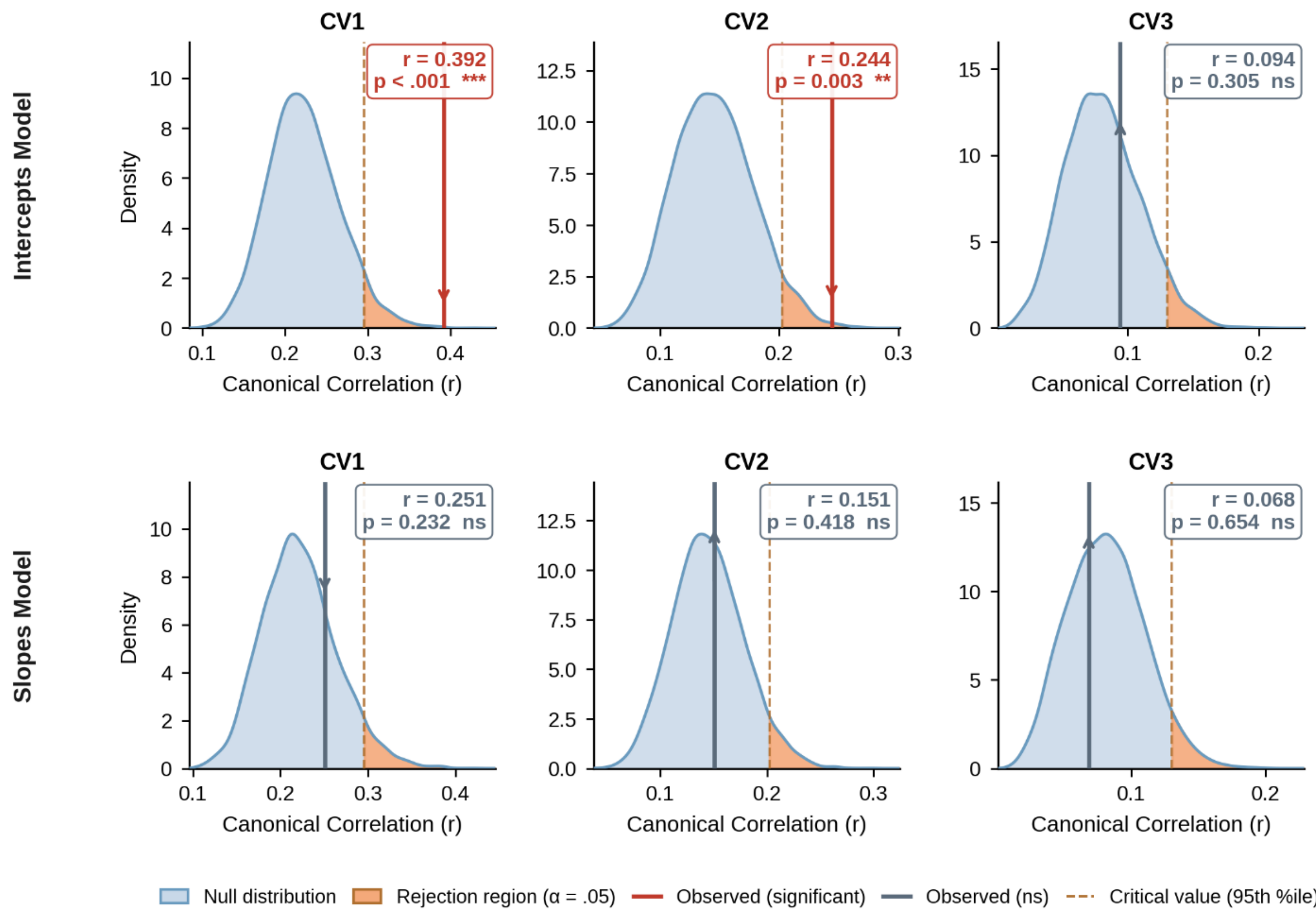

Note. Permutation-Based Null Distributions for Canonical Correlation Analysis. Each panel shows the null distribution of canonical correlations (*r*) generated from 5,000 permutations of the biomarker matrix Y (rows shuffled, stress-PC matrix X held fixed), separately for the intercepts model (top row) and slopes model (bottom row). The orange shaded region marks the upper 5% of each null distribution (rejection region, α=.05); the dashed vertical line indicates the corresponding critical value (95th percentile). The solid vertical line indicates the observed canonical correlation for each variate, with red denoting statistical significance and grey denoting non-significance. Permutation *p*-values reflect the proportion of null canonical correlations meeting or exceeding the observed value. Two canonical variates reached significance in the intercepts model (CV1: r=0.392, p<.001; CV2: r=0.244, p=.003); no variates reached significance in the slopes model (all *p*s>.23). *** p<.001; ** p<.01; ns=not significant.

10. Structural Imaging Sensitivity Analysis: Cortical Thickness and Surface Area

Supplemental Methods: Structural Imaging Sensitivity Analysis

To examine whether findings depended on the brain age gap (BAG) metric specifically, we conducted a pre-specified sensitivity CCA substituting BAG with two direct structural brain measures derived from the same T1-weighted scans: mean cortical thickness (CT; average of left and right hemisphere global thickness) and total cortical surface area (SA; sum of left and right hemisphere surface area), extracted from FreeSurfer's 34 Desikan-Killiany regions. Both CT and SA were age-residualized using the same OLS approach as BAG prior to inclusion. This analysis included N=190 participants with complete data on all seven biomarkers (mtDNA CN, telomere length, cortisol AUC, BMI, pubertal stage, CT, and SA) and the three stress PCs.

Supplemental Results: Structural Imaging Sensitivity Analysis

Prior to conducting the intercepts CCA, we examined bivariate associations between the cortical measures and BAG. Age-residualized CT was weakly negatively correlated with BAG (r=-0.156, p=.032, N=190), indicating modest overlap between thinner cortex and older-appearing brains. Age-residualized SA was uncorrelated with BAG (r=-0.014, p=.846). Critically, CT and SA were positively correlated with each other (r=0.450, p<.001).

The sensitivity CCA with age-residualized CT and SA replacing BAG yielded two statistically significant canonical variates (CV1: r=0.374, p=.016; CV2: r=0.285, p=.002; CV3: r=0.139, p=.133; N=190). The stress-biomarker configurations were consistent with the main analysis, with the deprivation-threat distinction preserved across CVs (see Supplemental Figure 5). CV1 was defined by cumulative stress and trauma (PC1 loading = +0.961) co-occurring with higher BMI (+0.735), earlier pubertal development (+0.541), and, in the opposite direction, lower cortical thickness (-0.351) and surface area (-0.464). CV2 was defined by low parental support (PC2 loading = +0.842) and neighborhood disadvantage (+0.538) co-occurring with lower mtDNA copy number (-0.721) and shorter telomere length (-0.553), with cortical measures

loading near zero (CT=+0.034, SA=+0.284). Notably, the CV order was reversed relative to the main analysis (threat emerging as CV1 rather than CV2), likely reflecting the reduced dimensionality of the biomarker space after removing BAG. Taken together, these findings indicate that the core stress-biology patterns identified in the primary CCA are not artifacts of the BAG composite and generalize to direct structural brain measures.

Figure S5: Structural Imaging Sensitivity Analysis

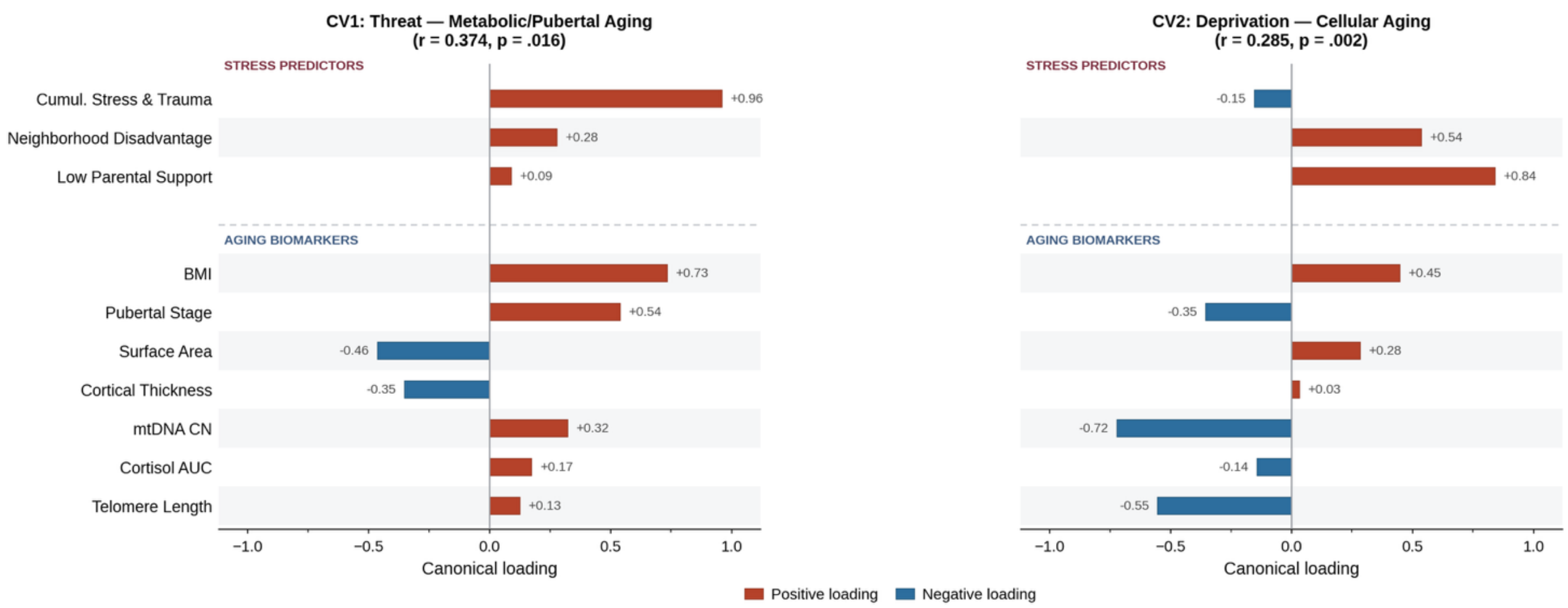

Note. Structural Sensitivity CCA: Canonical Loadings with Cortical Thickness and Surface Area Replacing Brain Age Gap (N=190). Canonical loadings from a sensitivity canonical correlation analysis (CCA) in which the Brain Age Gap (BAG) was replaced by two direct structural brain measures: mean cortical thickness (CT; average of left and right hemisphere global thickness) and total cortical surface area (SA; sum of left and right hemisphere areas), both age-residualized prior to analysis. The seven-biomarker set (mtDNA copy number, telomere length, cortisol AUC, BMI, pubertal stage, CT, SA) was entered against the three adversity principal components (PC1: Cumulative Stress & Trauma; PC2: Low Parental Support; PC3: Neighborhood Disadvantage) in N=190 participants with complete data. Two canonical variates reached statistical significance based on permutation testing (5,000 permutations): CV1 (r=0.374, p=.016) and CV2 (r=0.285, p=.002). CV1 recapitulates the threat-metabolic pattern from the primary analysis, with cumulative stress co-occurring with higher BMI and earlier pubertal stage, and lower cortical thickness and surface area. CV2 recapitulates the deprivation-cellular aging pattern, with low parental support and neighborhood disadvantage co-occurring with lower mtDNA copy number and shorter telomere length; cortical measures load near zero on this variate (CT=0.03, SA=0.28). Loadings are sorted within each section by absolute magnitude. Bars reflect structure coefficients (variable-variate correlations); signs are oriented so that the primary adversity predictor is positive within each CV.

References

1. Ribbe D. Psychometric review of traumatic event screening instrument for children (TESI-C). In Measurement of Stress, Trauma, and Adaptation. 1996;Lutherville, MD: Sidran.

2. King LS, Graber MG, Colich NL, Gotlib IH. Associations of waking cortisol with DHEA and testosterone across the pubertal transition: Effects of threat-related early life stress. Psychoneuroendocrinology. 2020 May 1;115:104651. doi:10.1016/j.psyneuen.2020.104651

3. Rudolph KD, Hammen C, Burge D, Lindberg N, Herzberg D, Daley SE. Toward an interpersonal life-stress model of depression: The developmental context of stress generation. Development and Psychopathology. 2000 Jun;12(2):215–34. doi:10.1017/S0954579400002066

4. Chahal R, Miller JG, Yuan JP, Buthmann JL, Gotlib IH. An exploration of dimensions of early adversity and the development of functional brain network connectivity during adolescence: Implications for trajectories of internalizing symptoms. Development and Psychopathology. 2022 May;34(2):557–71. doi:10.1017/S0954579421001814

5. King LS, Graber MG, Colich NL, Gotlib IH. Associations of waking cortisol with DHEA and testosterone across the pubertal transition: Effects of threat-related early life stress. Psychoneuroendocrinology. 2020 May;115:104651. doi:10.1016/j.psyneuen.2020.104651 PubMed PMID: 32199287; PubMed Central PMCID: PMC7183417.

6. Kantor GK, Holt MK, Mebert CJ, Straus MA, Drach KM, Ricci LR, et al. Development and Preliminary Psychometric Properties of the Multidimensional Neglectful Behavior Scale-Child Report. Child Maltreat. 2004 Nov 1;9(4):409–28. doi:10.1177/1077559504269530

7. Reitman D, Rhode PC, Hupp SDA, Altobello C. Development and Validation of the Parental Authority Questionnaire – Revised. Journal of Psychopathology and Behavioral Assessment. 2002 Jun 1;24(2):119–27. doi:10.1023/A:1015344909518

8. Hooley JM, Teasdale JD. Predictors of relapse in unipolar depressives: Expressed emotion, marital distress, and perceived criticism. Journal of Abnormal Psychology. 1989;98(3):229–35. doi:10.1037/0021-843X.98.3.229

9. Capaldi DM, Patterson GR. Psychometric properties of fourteen latent constructs from the Oregon Youth Study. New York, NY, US: Springer-Verlag Publishing; 1989. vi, 287 p. (Psychometric properties of fourteen latent constructs from the Oregon Youth Study). doi:10.1007/978-1-4612-3562-0

10. Barber BK. Parental psychological control: revisiting a neglected construct. Child development. 1996 Dec;67(6). PubMed PMID: 9071782.

11. Robinson CC, Mandleco B, Olsen SF, Hart CH. Authoritative, Authoritarian, and Permissive Parenting Practices: Development of a New Measure. Psychol Rep. 1995 Dec 1;77(3):819–30. doi:10.2466/pr0.1995.77.3.819

12. Robinson C, Mandleco B, Olson S, Hart C. The parenting styles and dimensions questionnaire (PSDQ). In: Handbook of family measurement … [Internet]. 2001 [cited 2024 Nov 11]. p. 319–21. Available from:

https://www.academia.edu/13707474/The_parenting_styles_and_dimensions_questionnaire_PSDQ_

13. Achenbach TM. Achenbach system of empirically based assessment (ASEBA). The encyclopedia of clinical psychology. 2014;1–8.

14. OEHHA. CalEnviroScreen 4.0 [text] [Internet]. 2021 [cited 2026 Mar 29]. Available from: https://oehha.ca.gov/calenviroscreen/report/calenviroscreen-40

15. Zimet GD, Dahlem NW, Zimet SG, Farley GK. The Multidimensional Scale of Perceived Social Support. Journal of Personality Assessment. 1988 Mar 1;52(1):30–41. doi:10.1207/s15327752jpa5201_2

16. Wongpakaran T, Wongpakaran N, Ruktrakul R. Reliability and Validity of the Multidimensional Scale of Perceived Social Support (MSPSS): Thai Version. Clin Pract Epidemiol Ment Health. 2011 Oct 26;7:161–6. doi:10.2174/1745017901107010161 PubMed PMID: 22114620; PubMed Central PMCID: PMC3219878.

17. Sanders B, Becker-Lausen E. The measurement of psychological maltreatment: early data on the Child Abuse and Trauma Scale. Child Abuse Negl. 1995 Mar;19(3):315–23. doi:10.1016/s0145-2134(94)00131-6 PubMed PMID: 9278731.

18. Kind AJH, Jencks S, Brock J, Yu M, Bartels C, Ehlenbach W, et al. Neighborhood socioeconomic disadvantage and 30-day rehospitalization: a retrospective cohort study. Ann Intern Med. 2014 Dec 2;161(11):765–74. doi:10.7326/M13-2946 PubMed PMID: 25437404; PubMed Central PMCID: PMC4251560.

19. Singh GK. Area Deprivation and Widening Inequalities in US Mortality, 1969–1998. Am J Public Health. 2003 Jul;93(7):1137–43. doi:10.2105/ajph.93.7.1137 PubMed PMID: 12835199; PubMed Central PMCID: PMC1447923.

20. Humphreys KL, Sisk LM, Manczak EM, Lin J, Gotlib IH. Depressive Symptoms Predict Change in Telomere Length and Mitochondrial DNA Copy Number Across Adolescence. J Am Acad Child Adolesc Psychiatry. 2020 Dec;59(12):1364-1370.e2. doi:10.1016/j.jaac.2019.09.031 PubMed PMID: 31628984; PubMed Central PMCID: PMC7160006.

21. Berens A, LeMoult J, Kircanski K, Gotlib IH. ADHD symptoms and diurnal cortisol in adolescents: The importance of comorbidities. Psychoneuroendocrinology. 2023 Feb;148:105990. doi:10.1016/j.psyneuen.2022.105990 PubMed PMID: 36462296; PubMed Central PMCID: PMC10193285.

22. King LS, Colich NL, LeMoult J, Humphreys KL, Ordaz SJ, Price AN, et al. The impact of the severity of early life stress on diurnal cortisol: The role of puberty. Psychoneuroendocrinology. 2017 Mar;77:68–74. doi:10.1016/j.psyneuen.2016.11.024 PubMed PMID: 28024271; PubMed Central PMCID: PMC5336485.

23. Yuan JP, Borchers LR, Lee Y, Buthmann JL, Coury SM, Joachimsthaler J, et al. The effects of the COVID-19 pandemic on neurobiological functioning in adolescents. Transl Psychiatry. 2025 Aug 12;15(1):276. doi:10.1038/s41398-025-03485-2

24. Pruessner JC, Kirschbaum C, Meinlschmid G, Hellhammer DH. Two formulas for computation of the area under the curve represent measures of total hormone concentration versus time-dependent change. Psychoneuroendocrinology. 2003 Oct 1;28(7):916–31. doi:10.1016/S0306-4530(02)00108-7

25. Miller JG, Ho TC, Humphreys KL, King LS, Foland-Ross LC, Colich NL, et al. Early Life Stress, Frontoamygdala Connectivity, and Biological Aging in Adolescence: A Longitudinal Investigation. Cereb Cortex. 2020 Jun 1;30(7):4269–80. doi:10.1093/cercor/bhaa057

26. Cawthon RM. Telomere measurement by quantitative PCR. Nucleic Acids Res. 2002 May 15;30(10):e47. doi:10.1093/nar/30.10.e47 PubMed PMID: 12000852; PubMed Central PMCID: PMC115301.

27. Drobinin V, Van Gestel H, Helmick CA, Schmidt MH, Bowen CV, Uher R. The Developmental Brain Age Is Associated With Adversity, Depression, and Functional Outcomes Among Adolescents. Biological Psychiatry: Cognitive Neuroscience and Neuroimaging. 2022 Apr 1;7(4):406–14. doi:10.1016/j.bpsc.2021.09.004

28. Gotlib IH, Miller JG, Borchers LR, Coury SM, Costello LA, Garcia JM, et al. Effects of the COVID-19 Pandemic on Mental Health and Brain Maturation in Adolescents: Implications for Analyzing Longitudinal Data. Biol Psychiatry Glob Open Sci. 2022 Dec 1;3(4):912–8. doi:10.1016/j.bpsgos.2022.11.002 PubMed PMID: 36471743; PubMed Central PMCID: PMC9713854.

29. Marshall WA, Tanner JM. Variations in pattern of pubertal changes in girls. Arch Dis Child. 1969 Jun;44(235):291–303. doi:10.1136/adc.44.235.291 PubMed PMID: 5785179; PubMed Central PMCID: PMC2020314.

30. Bond L, Clements J, Bertalli N, Evans-Whipp T, McMorris BJ, Patton GC, et al. A comparison of self-reported puberty using the Pubertal Development Scale and the Sexual Maturation Scale in a school-based epidemiologic survey. J Adolesc. 2006 Oct;29(5):709–20. doi:10.1016/j.adolescence.2005.10.001 PubMed PMID: 16324738.

31. Morris NM, Udry JR. Validation of a self-administered instrument to assess stage of adolescent development. J Youth Adolesc. 1980 Jun;9(3):271–80. doi:10.1007/BF02088471 PubMed PMID: 24318082.

32. McLaughlin KA, Sheridan MA, Lambert HK. Childhood Adversity and Neural Development: Deprivation and Threat as Distinct Dimensions of Early Experience. Neurosci Biobehav Rev. 2014 Nov;47:578–91. doi:10.1016/j.neubiorev.2014.10.012 PubMed PMID: 25454359; PubMed Central PMCID: PMC4308474.

33. McLaughlin KA, Sheridan MA. Beyond Cumulative Risk: A Dimensional Approach to Childhood Adversity. Current directions in psychological science. 2016 Aug;25(4):239. doi:10.1177/0963721416655883 PubMed PMID: 27773969.

34. Barcikowski RS, Stevens JP. A Monte Carlo Study of the Stability of Canonical Correlations, Canonical Weights and Canonical Variate-Variable Correlations. Multivariate Behav Res. 1975 Jul 1;10(3):353–64. doi:10.1207/s15327906mbr1003_8 PubMed PMID: 26829636.

35. Winkler AM, Renaud O, Smith SM, Nichols TE. Permutation inference for canonical correlation analysis. Neuroimage. 2020 Oct 15;220:117065. doi:10.1016/j.neuroimage.2020.117065 PubMed PMID: 32603857; PubMed Central PMCID: PMC7573815.
